# Predicting post-stroke cognitive impairment using acute CT neuroimaging: A systematic review and meta-analysis

**DOI:** 10.1177/17474930211045836

**Published:** 2021-09-29

**Authors:** Emily L Ball, Rachel Sutherland, Charlotte Squires, Gillian E Mead, Dorota Religa, Erik Lundström, Joshua Cheyne, Joanna M Wardlaw, Terence J Quinn, Susan D Shenkin

**Affiliations:** 1 Centre for Clinical Brain Sciences, University of Edinburgh, Edinburgh, UK; 2 NHS Lothian, Edinburgh, UK; 3 Geriatric Medicine, Usher Institute, University of Edinburgh, Edinburgh, UK; 4 Division of Clinical Geriatrics, Karolinska Institutet, Stockholm, Sweden; 5 Department of Neuroscience, Neurology, Uppsala University, Uppsala, Sweden; 6 Centre for Clinical Brain Sciences, UK Dementia Research Institute, University of Edinburgh, Edinburgh, UK; 7 Institute of Cardiovascular and Medical Sciences, University of Glasgow, Glasgow, UK

**Keywords:** Stroke, cognitive impairment, dementia, neuroimaging, computed tomography

## Abstract

**Background:**

Identifying whether acute stroke patients are at risk of cognitive decline could improve prognostic discussions and management. Structural computed tomography neuroimaging is routine in acute stroke, and may identify those at risk of post-stroke dementia or post-stroke cognitive impairment (PSCI).

**Aim:**

To systematically review the literature to identify which stroke or pre-stroke features on brain computed tomography scans, performed at the time of stroke, are associated with post-stroke dementia or PSCI.

**Summary of review:**

We searched electronic databases to December 2020. We included studies reporting acute stroke brain computed tomography, and later diagnosis of a cognitive syndrome. We created summary estimates of size of unadjusted association between computed tomography features and cognition. Of 9536 citations, 28 studies (41 papers) were eligible (N = 7078, mean age 59.8–78.6 years). Cognitive outcomes were post-stroke dementia (10 studies), PSCI (17 studies), and one study analyzed both. Fifteen studies (N = 2952) reported data suitable for meta-analyses. White matter lesions (WML) (six studies, N = 1054, OR = 2.46, 95% CI = 1.25–4.84), cerebral atrophy (four studies, N = 558, OR = 2.80, 95% CI = 1.21–6.51), and pre-existing stroke lesions (three studies, N = 352, OR = 2.38, 95% CI = 1.06–5.32) were associated with post-stroke dementia. WML (four studies, N = 473, OR = 3.46, 95% CI = 2.17–5.52) were associated with PSCI. Other computed tomography features were either not associated with cognitive outcome, or there were insufficient data.

**Conclusions:**

Cognitive impairment following stroke is of great concern to patients and carers. Features seen on visual assessment of acute stroke computed tomography brain scans are strongly associated with cognitive outcomes. Clinicians should consider when and how this information should be discussed with stroke survivors.

## Introduction

Cognitive decline and dementia are common following stroke.^1^ According to the Stroke Association-James Lind Alliance 2021 survey, patients and carers are more concerned about cognitive impairment after stroke than having another stroke.^2^ Identifying patients at high risk of persisting cognitive issues following stroke could allow for targeted follow-up, and assist discussions around prognosis. There are also research implications for identifying patients at risk of dementia, including creating enriched populations (i.e. people who are most at risk of subsequent dementia or cognitive impairment) for future studies of possible cognitive interventions.

The cognitive problems that follow stroke have various labels. In this review, we use the terms, post-stroke dementia (PSD – defined as a diagnosis of any type of dementia following stroke) and post-stroke cognitive impairment (PSCI – encompassing all severities of cognitive impairment).^3^ Classical risk factors associated with PSD include low education, atrial fibrillation, and recurrent stroke.^1^

CT or MRI neuroimaging features have also shown associations with PSD/PSCI.^4,5^ A systematic review describing white matter lesions (WML) and PSCI reported an approximate doubling of risk between WML and PSCI/PSD.^5^ Another review, across mixed stroke populations, reported that global and medial temporal lobe atrophy were consistently associated with PSCI.^4^ These reviews highlight that neuroimaging features are associated with cognitive outcomes, included imaging on both CT and MRI, taken up to several months after the acute stroke.

In acute stroke, the main reason for neuroimaging is to diagnose the cause of the stroke, which in turn determines hyperacute treatment. Although MRI produces a higher resolution image, structural CT neuroimaging is faster, cheaper, and remains the international standard neuroimaging technique.

Our systematic review focuses on the prognostic utility of routine CT performed at the time of stroke. We included studies of TIA, ischemic, and hemorrhagic stroke, and investigated whether pre-existing stroke features and acute stroke lesions are associated with PSD and PSCI.

## Methods

### Protocol and registration

The protocol was registered on PROSPERO (CRD42019128677) and is reported according to PRISMA guidelines.^6^

### Eligibility criteria

We included observational studies and clinical trials in any language which: included patients with first or recurrent stroke/TIA, performed structural CT neuroimaging at the point of stroke (i.e. within the acute stroke period, defined as 0–30 days from index stroke), and assessed cognition using validated cognitive assessments and/or diagnosed dementia using recognized diagnostic criteria, at least three months after the stroke.

### Information sources

The search strategy was designed in consultation with an information specialist and clinicians with expertise in stroke, neuroimaging, and evidence synthesis. We searched electronic databases Embase (OVID), MEDLINE (OVID), PsycINFO (EBSCO), and Cochrane Central Register of controlled Trials (CENTRAL) from inception to December 2020. Each search strategy included controlled vocabulary and keywords combining key concepts of: stroke, dementia/cognitive impairment, neuroimaging, and study type (Supplement 1). Published papers, and abstracts that presented quantitative data, were eligible for inclusion. We hand-searched references of included articles and relevant reviews. We contacted study authors if fundamental details were unclear in the published paper, and if they did not respond, we excluded the study.

### Study selection

Studies were imported and de-duplicated using Covidence software (Veritas Health Innovation Ltd).^7^ Titles, abstracts, and full text articles were independently screened by two reviewers, disagreements were resolved by consensus or by a third author. We excluded studies which did not distinguish whether neuroimaging features were identified on CT or MRI.

### Data collection process

We extracted data based on a modified version of the CHARMS-PF checklist (CHecklist for critical Appraisal and data extraction for systematic Reviews of prediction Modelling Studies, tailored to Prognostic Factor studies).^8^ Half of studies were independently extracted by two reviewers and disagreements were resolved by consensus or by a third reviewer. As disagreements were minor and infrequent, we were satisfied that the remainder could be single assessed. Where multiple papers reported the same cohort, data were extracted from the paper with the most complete data relating to the primary outcome.

### Data extraction

We extracted data including: study setting, participant inclusion and exclusion criteria, demographic information including vascular risk factors, length of follow-up, method of assessing dementia or cognitive function, and cognitive outcome (at latest time point). We extracted raw data and effect sizes relating to structural CT neuroimaging features, including acute stroke features and pre-existing stroke features (Supplement 2).

### Quality assessment

We assessed the quality of the included studies by using the Quality in Prognostic factor Studies (QUIPS) tool.^8^ We used Grading of Recommendations Assessment, Development and Evaluation (GRADE) methods to describe confidence in the summary results based on: risk of bias, inconsistency, imprecision, publication bias, and size of association.^9^

### Synthesis of neuroimaging features

CT neuroimaging features were pre-specified as follows: atrophy, WML, pre-existing stroke lesions (silent brain infarcts, old stroke lesions), pathological stroke type, acute stroke features (location, size, number of lesions, swelling), and combinations of neuroimaging features. We accepted the categorization used in the parent study. We used a harvest plot approach for data visualization.^10^ Data relating to acute stroke lesions and combinations of neuroimaging features were too heterogeneous for quantitative or semi-quantitative synthesis and were described as a narrative.

### Meta-analyses

We included studies in our meta-analyses if they reported odds ratios (OR) that compared presence versus absence of the neuroimaging feature, or provided sufficient data to derive the OR. We log transformed the OR and confidence intervals (CI) and performed random-effects meta-analyses using the inverse-variance method. We quantified heterogeneity with *I*^2^. We performed separate analyses for studies which reported PSD or PSCI outcomes. These analyses used RStudio software.

We limited our analyses to unadjusted data because there were insufficient data available to pool adjusted effect sizes. We created summary effect ORs for cerebral atrophy, WML, pre-existing stroke lesions, and pathological stroke type. Because studies used a variety of methods to grade severity of atrophy or WML, we dichotomized into presence or absence of these features.

### Sensitivity analyses

We conducted three sensitivity analyses exploring effect of: length of follow-up, limiting to studies at least six months post-stroke (planned sensitivity analysis), ischemic stroke type (post-hoc analysis), pre-stroke cognitive impairment (post-hoc analysis). The data did not allow for our other planned analyses (severity of cognitive impairment, treatment).

## Results

**Figure 1.**
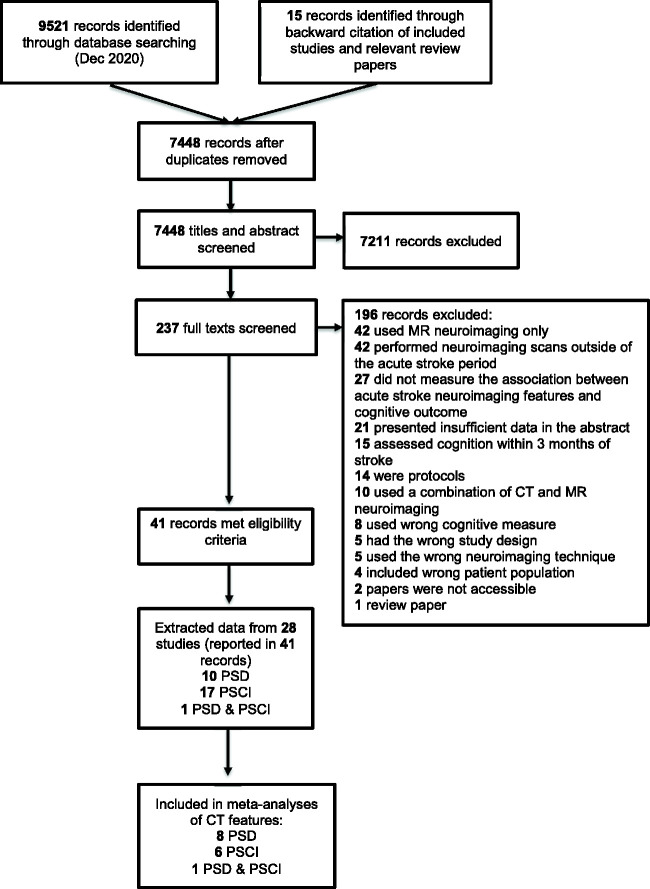
Study selection flow diagram.

From 9536 records, 28 studies^11–38^ (described in 41 papers, references provided in the Supplementary File) were eligible for inclusion (N = 7078, mean age range 59.8–78.6 years, 18–54% female) (Figure 1).

### Study characteristics

Studies included: ischemic and hemorrhagic strokes (12 studies),^12–14,17,19–22,25,32,36,38^ ischemic stroke and TIA (one study),^33^ ischemic strokes only (10 studies),^11,16,18,23,26,29–31,35,37^ hemorrhagic strokes only (two studies)^15,28^ (Supplement 3–5). Two studies only recruited lacunar infarcts^24,27^ and one study only recruited supratentorial infarcts.^34^ CT scans were performed: at admission/as soon as possible (10 studies),^11,17,19,21,24,28,30,35,37,38^ within 48 h (six studies),^15,16,22,29,31,33^ within one week (eight studies),^13,18,20,23,26,32,34,36^ within two weeks (three studies),^12,25,27^ within 0–30 days (one study).^14^

### Cognitive follow-up

Length of follow-up for PSD and/or PSCI ranged from three months to six years after stroke (Supplement 5). The primary cognitive outcomes were PSD (10 studies; prevalence ranging from 11 to 50%),^14–16,22–25,27,28,36^ PSCI (17 studies; prevalence ranging from 8 to 80%),^11–13,17–21,26,29,30,32–35,37,38^ and one study analyzed both PSD and PSCI separately (prevalence 10% and 59%, respectively).^31^ A variety of measures were used for diagnosing dementia and assessing cognition (Supplement 5).

### CT neuroimaging features

A broad range of CT features were reported: atrophy (14 studies),^12,13,18,22–26,28,31–34,36^ WML (18 studies),^13,15,20–28,31–34,36–38^ pre-existing stroke lesions (10 studies),^13,16,18,20–22,25,31–33^ pathological stroke type (seven studies),^14,20,22,25,32,36,38^ acute stroke features (23 studies),^11–15,18–24,26,28–36,38^ combinations of neuroimaging features (five studies).^13,17,19,25,31^ A comprehensive list of all CT neuroimaging features reported in the studies are presented in Supplement 6 to 11.

### Harvest plot

**Figure 2.**
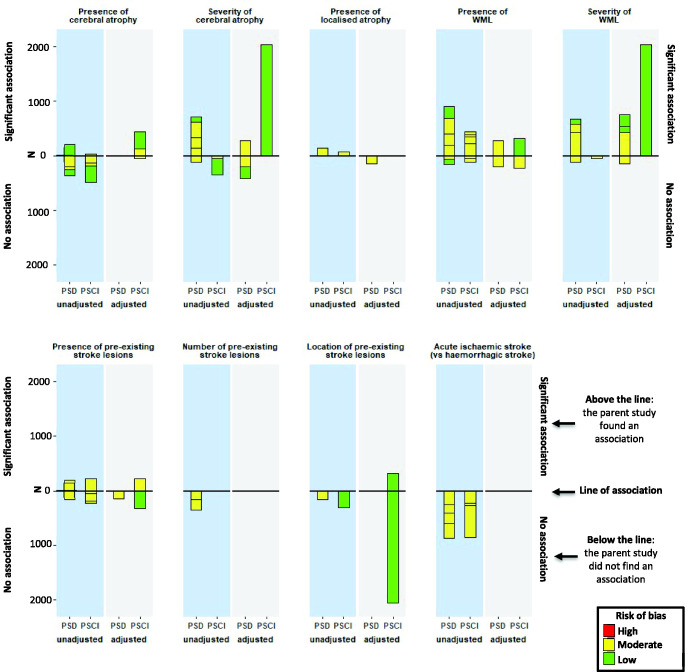
Harvest plot visualizing association between CT neuroimaging feature and subsequent development of post-stroke dementia or post-stroke cognitive impairment. This harvest plot visualizes whether studies found a statistically significant association or not between the CT features of interest and PSD or PSCI following unadjusted or adjusted analyses. Each unit represents a study, if the unit lies above the line of association the individual study found a statistically significant association, if the unit lies below the line the study did not find a statistically significant association. The y-axis represents the number of patients in each study, the size of each unit relates to the study sample size. The left hand column (pale blue) shows unadjusted associations between neuroimaging features and cognitive outcome (i.e. the study did not adjust for other risk factors of post-stroke dementia). The right hand column (light grey) shows findings from studies that adjusted for other risk factors of post-stroke dementia. The color of each unit depicts the overall risk of bias for each study (green = low; yellow = moderate; red = high). When studies graded the severity of cerebral atrophy or WML, data were dichotomized into presence or absence of these features. PSCI: post-stroke cognitive impairment; PSD: post-stroke dementia; WML: white matter lesions.

Data from 11 studies reporting PSD^14–16,22–25,27,28,31,36^ and 12 studies reporting PSCI^12,13,18,20,21,26,31–34,37,38^ were included in the harvest plot (Figure 2). The remaining six studies measured acute stroke features (e.g. lesion location, lesion size, number of lesions, edema), or combinations which were too heterogeneous to combine.^11,17,19,29,30,35^

### Risk of bias

We considered no studies to have an overall high risk of bias, 21 studies as moderate risk^11,12,14–17,19–22,24–27,31,32,34–38^ and seven studies as low risk^13,18,23,28–30,33^ (Supplement 12).

### Atrophy

Fourteen (N = 4368) studies measured atrophy (Figure 2).^12,13,18,22–26,28,31–34,36^ Severity of atrophy was associated with PSD (unadjusted analysis) and PSCI (adjusted analysis), but there was no clear association between presence of cerebral atrophy or localized atrophy and PSD or PSCI. Two studies also reported measures of atrophy which were too heterogeneous to include in the harvest plot. One study found no association between localized and generalized cerebral atrophy and PSCI (RR = 0.78, 95% CI = 0.48–1.29, unadjusted).^18^ The other study measured various indices of ventricular and cortical atrophy, and found an association with PSCI in four out of the six measures (Supplement 6).^34^

**Figure 3.**
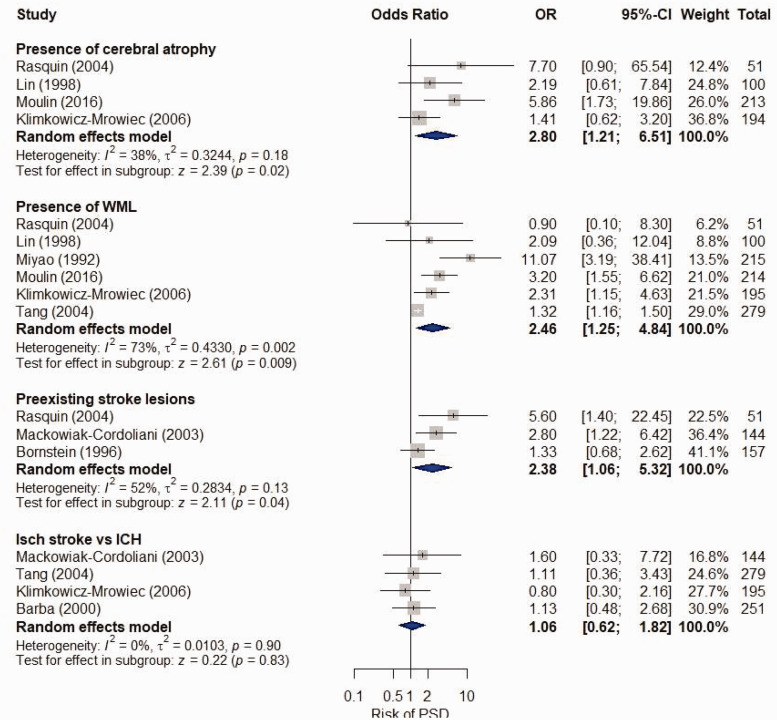
Unadjusted meta-analysis of CT features associated with post-stroke dementia (PSD). Box size proportionate to weight of study in meta-analysis (using the inverse-variance method). ICH: intracerebral hemorrhage; Isch: ischemic; PSD: post-stroke dementia; WML: white matter lesions.

**Figure 4.**
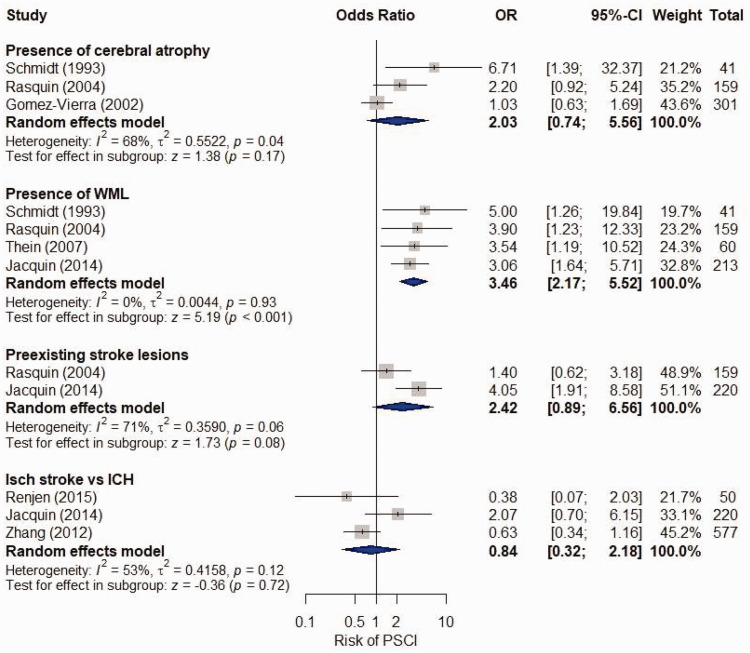
Unadjusted meta-analysis of CT features associated with post-stroke cognitive impairment (PSCI). Box size proportionate to weight of study in meta-analysis (using the inverse-variance method) (Source: Schmidt et al.^34^ reported presence of moderately severe ventricular atrophy). Schmidt (1993) reported presence of moderately severe ventricular atrophy. ICH: intracerebral hemorrhage; Isch: ischemic; PSCI: post-stroke cognitive impairment; WML: white matter lesions.

Six studies had suitable data for meta-analysis. The presence of cerebral atrophy was associated with PSD (four studies, N = 558, OR = 2.80, 95% CI = 1.21–6.51; *I*^2 ^= 38%, P = .18)^22,23,28,31^ but not PSCI (three studies, N = 501, OR = 2.03, 95% CI = 0.74-5.56; *I*^2 ^= 68%, P = .04)^18,31,34^ (Figures 3 and 4). We have low to moderate confidence in these results (Supplement 13). Sensitivity analyses are presented in Supplement 14 and 15.

### WML

Eighteen (N = 5521) studies reported WML (Figure 2).^13,15,20–28,31–34,36–38^ Presence of WML was associated with PSD and PSCI (unadjusted analysis), severity of WML was associated with PSD (unadjusted and adjusted analysis) and PSCI (adjusted analysis). As well as measuring overall WML, one study measured subcortical WML and periventricular changes and found associations with PSD in unadjusted but not adjusted analysis (Supplement 7).^23^ One study measured frequency and degree of WML and did not find an association after adjusted analysis (Supplement 7).^24^

In nine studies, presence of WML was associated with PSD (six studies, N = 1054, OR = 2.46, 95% CI = 1.25–4.84; *I*^2 ^= 73%, P = .002)^22,23,27,28,31,36^ and PSCI (four studies, N = 473, OR = 3.46, 95% CI = 2.17-5.52; *I*^2 ^= 0%, P = .93)^20,31,34,37^ (Figures 3 and 4). We have low to moderate confidence in these results (Supplement 13). Sensitivity analyses are presented in Supplement 14 and 15.

### Pre-existing stroke lesions

Ten (N = 3872) studies reported pre-existing stroke lesions (Figure 2).^13,16,18,20–22,25,31–33^ There was no clear association between presence, number, and location of pre-existing stroke lesions and PSD/PSCI after unadjusted or adjusted analyses (when data were available). One of these studies performed adjusted analyses and reported that pre-existing bilateral and subcortical infarcts were associated with PSCI but not thalamic infarcts (Supplement 8).^33^ Another study reported location of old infarcts (striatocapsular, border zone, lacunar) and found either no association, or an association with only one of their cognitive tests (Supplement 8).^13^

In our meta-analyses of five studies, pre-existing stroke lesions were associated with PSD (three studies, N = 352, OR = 2.38, 95% CI = 1.06–5.32; *I*^2 ^= 52%, P = .13)^16,25,31^ but not PSCI (two studies, N = 379, OR = 2.42, 95% CI = 0.89–6.56; *I*^2 ^= 71%, P = .06) (Figures 3 and 4).^20,31^ We are moderately confident in these results (Supplement 13). Sensitivity analyses are presented in Supplement 14 and 15.

### Pathological stroke type

Twelve studies recruited patients with ischemic and hemorrhagic stroke.^12–14,17,19–22,25,32,36,38^ Seven (N = 1716) reported sufficient data assessing the association between pathological stroke type and cognitive outcome (Figure 2).^14,20,22,25,32,36,38^ There was no association between acute ischemic stroke and PSD or PSCI following unadjusted analysis (Supplement 9). No adjusted data were available. On meta-analysis, acute ischemic stroke was not associated with PSD (four studies, N = 869, OR = 1.06, 95% CI = 0.62–1.82; *I*^2 ^= 0%, P = .90)^14,22,25,36^ or PSCI (three studies, N = 847, OR = 0.84, 95% CI = 0.32-2.18; *I*^2 ^= 53%, P = .12)^20,32,38^ (Figures 3 and 4). We have low to moderate confidence in these results (Supplement 13).

### Acute stroke features

Twenty-three studies (N = 6273) reported acute stroke features.^11–15,18–24,26,28–36,38^ Studies reported highly heterogeneous features which we grouped into location, size, number of lesions, and swelling (Supplement 10). Evidence was heterogeneous; however, in general, there were no clear predictors of PSD or PSCI.

### Combinations of CT features

Five studies (N = 2901) reported combinations of neuroimaging features (Supplement 11).^13,17,19,25,31^ One study found no association between small vessel disease (SVD, not defined by study) and PSD following unadjusted analysis.^25^ Another study measured SVD (severe WML, severe atrophy, old lacunar infarcts/lacunes) but only found an association with one of their three cognitive tests following adjusted analysis.^13^ When the same study measured brain frailty (WML, cerebral atrophy, old vascular lesions/infarcts), they found an association with all cognitive measures.^13^ One study found an association between silent brain infarcts and WML, and PSD; however, the presence of these two features were not associated with PSCI.^31^ One study measured cortical atrophy and WML, and found an association with PSCI.^19^ One measured lesions defined as mainly lacunes and WML and found an association with PSCI.^17^

### Demographic and vascular risk factors

Demographic and vascular risk factors had a weaker association with cognitive outcome, compared to the CT features that were associated with PSD or PSCI (Supplement 16 and 17).

## Discussion

This systematic review and meta-analysis of 28 studies including 7078 patients found that CT features, visible at the point of stroke, were associated with increased risk of both PSD and PSCI. In our meta-analysis, we found that presence of atrophy, WML, and pre-existing stroke lesions were associated with a two- to three-fold increase in the odds of developing PSD. WML were also associated with a threefold increase in the odds of developing PSCI. We did not find an association between presence of atrophy, pre-existing stroke lesions and PSCI; however, fewer studies were included in the meta-analysis of these CT features and these studies followed up patients for a shorter timeframe than the studies which diagnosed dementia. We have low to moderate confidence in these findings. Seven studies had low risk of bias. Generally, acute stroke lesions (location, size, number of lesions, swelling) were not associated with cognitive outcome; however, they were highly heterogeneous. There was limited evidence for combinations of neuroimaging features.

This is the first systematic review to consider *all* routinely accessible CT brain imaging features, including acute and pre-stroke features, at the time of stroke, to predict PSD or PSCI. Previous reviews had also found that features including atrophy and WML are associated with cognitive impairment and dementia following a stroke.^4,5^ However, these reviews primarily focused on the association between pre-existing neuroimaging features and cognitive outcome, rather than also including acute stroke features, and included studies which performed neuroimaging up to several months after the stroke, which is not routine clinical practice. These reviews also incorporated neuroimaging features identified on both CT and MRI scans, but acute MRI is not routinely performed in most hospitals. Our systematic review focused on the clinical utility of CT performed at the time of stroke, reflecting clinical practice.

### Strengths and limitations

Our review has several strengths, we used robust data extraction (CHARMS-PF) and quality assessment tools (QUIPS) tailored for prognostic factor studies, and framed our results using the GRADE approach.^8^ We included studies and abstracts in all languages and contacted study authors for additional information, which allowed us to include a further three studies.

There were limitations in our review method and in the primary papers. There were challenges with pooling data from individual studies due to limited reporting and heterogeneity of methods for cognitive assessment and CT acquisition. Severity of CT features are likely to influence cognitive outcome after stroke. Due to heterogeneous methods used to report CT scans, we dichotomized severity of atrophy and WML into presence versus absence of these features in our meta-analyses. Some of this heterogeneity could be improved by the use of accepted criteria for reporting neuroimaging scans (e.g. Standards for ReportIng Vascular changes on nEuroimaging (STRIVE)).^39^ We must also consider the interaction between neuroimaging features and other risk factors for PSD/PSCI. For example, severity of atrophy and WML are associated with older age.^40^ To identify the independent prognostic effect of CT features, they must be adjusted for other key risk factors of PSD. As shown by our harvest plot, only a limited number of included studies performed adjusted analysis.

The majority of included studies excluded patients who were unable to complete the relevant cognitive testing, a group of people who likely had cognitive issues or severe stroke. Several of the included studies did not report the length of time between stroke and brain scan and study authors had to be contacted. The length of time between stroke and brain scan will impact the CT features that are visible on the scan and should be clearly reported in imaging studies.^41^ With a few exceptions, many studies were small, exploratory studies, which measured multiple prognostic factors and had few cases of PSD or PSCI. Even with meta-analysis, we may have only had power to detect the largest effect sizes and important but more modest effects may have been missed.^8^

### Clinical and research implications

Cognitive issues following stroke are of great concern to patients with stroke.^2^ Identifying patients, at the point of stroke, who are at an increased risk of developing PSD could influence follow-up care. For example, presence of extensive WML and atrophy might trigger follow-up to review cognition. Understanding a patient’s risk of PSD could enable healthcare professionals to start a conversation with patients and their families about the possibility of future cognitive decline.

Further, prognostic studies which use standardized reporting of acute stroke CT scans and implement standardized neuropsychological tests are necessary to explore the usefulness of acute stroke neuroimaging for predicting cognitive issues following stroke. Data linkage studies, linking acute stroke neuroimaging scans to subsequent diagnosis of dementia could be used to identify prognostic factors of PSD.

## Conclusions

Acute stroke CT is a routinely performed investigation which is crucial in determining hyperacute treatment and could also be used to identify patients at high risk of post-stroke cognitive problems.

## Supplemental Material

sj-pdf-1-wso-10.1177_17474930211045836 - Supplemental material for Predicting post-stroke cognitive impairment using acute CT neuroimaging: A systematic review and meta-analysis Click here for additional data file. Supplemental material, sj-pdf-1-wso-10.1177_17474930211045836 for Predicting post-stroke cognitive impairment using acute CT neuroimaging: A systematic review and meta-analysis by Emily L Ball, Rachel Sutherland, Charlotte Squires, Gillian E Mead, Dorota Religa, Erik Lundström, Joshua Cheyne, Joanna M Wardlaw, Terence J Quinn and Susan D Shenkin in International Journal of Stroke
