# Supplementary material for: Predicting post-stroke cognitive impairment using acute CT neuroimaging: A systematic review and meta-analysis
Source: Int J Stroke. 2021 Sep 29;17(6):618–27. doi: 10.1177/17474930211045836 (PMC9260488; doi:10.1177/17474930211045836)
Supplement: sj-pdf-1-wso-10.1177_17474930211045836 - Supplemental material for Predicting post-stroke cognitive impairment using acute CT neuroimaging: A systematic review and meta-analysis [file sj-pdf-1-wso-10.1177_17474930211045836.pdf]

Supplementary Online Content

Supplement 1: Search strategy 2
Supplement 2: Data extraction form 8
Supplement 3: Study characteristics of included post-stroke dementia and post-stroke cognitive impairment studies 11
Supplement 4: Clinical risk factors of stroke population included in cognitive follow-up 14
Supplement 5: Cognitive follow-up of PSD and PSCI studies 19
Supplement 6: Atrophy 22
Supplement 7: White matter lesions 28
Supplement 8: Pre-existing stroke lesions 33
Supplement 9: Pathological stroke type 37
Supplement 10: Acute stroke features 39
Supplement 11: Combinations of neuroimaging features 54
Supplement 12: Risk of bias assessment 56
Supplement 13: GRADE assessment 57
Supplement 14: Sensitivity Analysis PSD 59
Supplement 15: Sensitivity Analysis PSCI 60
Supplement 16: Meta-analysis of risk factors associated with PSD (unadjusted prognostic factors) 61
Supplement 17: Meta-analysis of risk factors associated with PSCI (unadjusted prognostic factors) 62
References 63

This supplementary material has been provided by the authors to give readers additional information about their work.

Supplement 1: Search strategy

Medline

Stroke

1. cerebrovascular disorders/ or exp basal ganglia cerebrovascular disease/ or brain ischemia/ or hypoxia-ischemia, brain/ or ischemic attack, transient/ or carotid artery diseases/ or carotid artery thrombosis/ or intracranial arterial diseases/ or cerebral arterial diseases/ or exp "intracranial embolism and thrombosis"/ or exp intracranial hemorrhages/ or exp stroke/
2. (stroke$ or apoplex$ or cerebral vasc$ or cerebrovasc$ or cva or transient isch$mic attack$ or tia).tw.
3. ((brain or cerebr$ or cerebell$ or hemispher$ or intracran$ or intracerebral or infratentorial or supratentorial or space-occupying) adj5 (isch?emi$ or infarct$ or thrombo$ or emboli$ or occlus$ or hypoxi$)).tw.
4. ((brain or cerebr$ or cerebell$ or hemispher$ or intracran$ or intracerebral or infratentorial or supratentorial or intraventricular) adj5 (h?emorrhag$ or h?ematoma$ or bleed$)).tw.
5. or/1-4

Dementia and cognitive impairment

6. exp dementia/ or memory disorders/ or neurocognitive disorders/ or cognition disorders/ or cognitive dysfunction/
7. dement$.tw.
8. ((attention or awareness or cognit$ or neuropsych$ or neurocognit$ or neurobehav$ or psycholog$ or memor$ or recall or think$) adj5 (declin$ or impair$ or domain$ or test$ or assess$ or function$ or batter$ or disorder$ or dysfunct$ or deficit$ or declin$ or abilit$ or problem$ or difficult$ or disturbance$ or disabilit$)).tw.
9. mental processes/ or cognition/ or awareness/ or cognitive reserve/ or executive function/ or learning/ or thinking/ or perception/ or memory/
10. exp psychological tests/
11. MMSE.tw.
12. or/6-11

Neuroimaging

13. diagnostic imaging/ or neuroimaging/ or multimodal imaging/
14. tomography, x-ray computed/
15. ((compute$ or cerebral or CAT or CT or brain) adj5 (imag$ or scan$ or neuroimag$ or tomogra$ or marker$ or feature$ or x-ray)).tw.
16. Magnetic resonance imaging/
17. (MRI or MRi or NMRI or NMRi).tw.
18. ((magn$ or resonance or MR or MTC or MT or NMR) adj5 (imag$ or scan$ or neuroimag$ or tomogra$ or marker$ or feature$)).tw.
19. or/13-18

Study type

20. prognosis/
21. observational study/
22. randomized controlled trial.pt.
23. controlled clinical trial.pt.
24. Epidemiologic Studies/
25. exp Case-Control Studies/
26. exp Cohort Studies/
27. (epidemiologic adj (study or studies)).ab,ti.
28. case control.ab,ti.
29. (cohort adj (study or studies)).ab,ti.
30. cohort analy$.ab,ti.
31. (follow up adj (study or studies)).ab,ti.
32. longitudinal.ab,ti.
33. retrospective$.ab,ti.
34. prospective$.ab,ti.
35. (observ$ adj3 (study or studies)).ab,ti.
36. or/20-35

Running the search

37. 5 and 12 and 19 and 36

EMBASE

Stroke

1. cerebrovascular disease/ or exp basal ganglion hemorrhage/ or brain ischemia/ or hypoxic ischemic encephalopathy/ or transient ischemic attack/ or carotid artery disease/ or carotid artery thrombosis/ or cerebral artery disease/ or brain embolism/ or occlusive cerebrovascular disease/ or cerebral sinus thrombosis/ or exp brain hemorrhage/ or hypophysis apoplexy/ or exp brain hematoma/ or epidural hematoma/ or exp cerebrovascular accident/ or exp brain infarction/
2. (stroke$ or apoplex$ or cerebral vasc$ or cerebrovasc$ or cva or transient isch$mic attack$ or tia).tw.
3. ((brain or cerebr$ or cerebell$ or hemispher$ or intracran$ or intracerebral or infratentorial or supratentorial or space-occupying) adj5 (isch?emi$ or infarct$ or thrombo$ or emboli$ or occlus$ or hypoxi$)).tw.
4. ((brain or cerebr$ or cerebell$ or hemispher$ or intracran$ or intracerebral or infratentorial or supratentorial or intraventricular) adj5 (h?emorrhag$ or h?ematoma$ or bleed$)).tw.
5. or/1-4

Dementia and cognitive impairment

6. exp dementia/ or memory disorder/ or disorders of higher cerebral function/ or cognitive defect/
7. dement$.tw.
8. ((attention or awareness or cognit$ or neuropsych$ or neurocognit$ or neurobehav$ or psycholog$ or memor$ or recall or think$) adj5 (declin$ or impair$ or domain$ or test$ or assess$ or function$ or batter$ or disorder$ or dysfunct$ or deficit$ or declin$ or abilit$ or problem$ or difficult$ or disturbance$ or disabilit$)).tw.
9. mental function/ or cognition/ or awareness/ or cognitive reserve/ or executive function/ or learning/ or thinking/ or perception/ or memory/
10. exp psychologic test/ or exp dementia assessment/ or exp cognition assessment/
11. MMSE.tw
12. or/6-11

Neuroimaging

13. diagnostic imaging/ or neuroimaging/ or multimodal imaging/
14. x-ray computed tomography/ or computer assisted tomography/ or high resolution computer tomography/
15. ((compute$ or cerebral or CAT or CT or brain) adj5 (imag$ or scan$ or neuroimag$ or tomogra$ or marker$ or feature$ or x-ray)).tw.
16. nuclear magnetic resonance imaging/
17. (MRI or MRi or NMRI or NMRi).tw.
18. ((magn$ or resonance or MR or MTC or MT or NMR) adj5 (imag$ or scan$ or neuroimag$ or tomogra$ or marker$ or feature$)).tw.
19. or/13-18

Study type

20. prognosis/
21. observational study/
22. randomized controlled trial.pt.
23. controlled clinical trial.pt.
24. exp case control study/
25. cohort analysis/
26. (epidemiologic adj (study or studies)).ab,ti.
27. case control.ab,ti.
28. (cohort adj (study or studies)).ab,ti.
29. cohort analy$.ab,ti.
30. (follow up adj (study or studies)).ab,ti.
31. longitudinal.ab,ti.
32. retrospective$.ab,ti.
33. prospective$.ab,ti.
34. (observ$ adj3 (study or studies)).ab,ti.
35. or/20-34

Running the search

36. 5 and 12 and 19 and 35

PsycINFO

Stroke

1. cerebrovascular disorders/ or cerebral ischemia/ or cerebral hemorrhage/ or cerebrovascular accidents/
2. (basal gangli$ h?morrhage$ or carotid artery disease$ or carotid artery thrombo$ or intracranial arter$ disease$ or cerebral arter$ disease$).tw.
3. (stroke$ or apoplex$ or cerebral vasc$ or cerebrovasc$ or cva or transient isch$mic attack$ or tia).tw.
4. ((brain or cerebr$ or cerebell$ or hemispher$ or intracran$ or intracerebral or infratentorial or supratentorial or space-occupying) adj5 (isch?emi$ or infarct$ or thrombo$ or emboli$ or occlus$ or hypoxi$)).tw.
5. ((brain or cerebr$ or cerebell$ or hemispher$ or intracran$ or intracerebral or infratentorial or supratentorial or intraventricular) adj5 (h?emorrhag$ or h?ematoma$ or bleed$)).tw.
6. or/1-5

Dementia and cognitive impairment

7. exp dementia/ or memory disorders/ or cognitive impairment/
8. dement$.tw.
9. ((attention or awareness or cognit$ or neuropsych$ or neurocognit$ or neurobehav$ or psycholog$ or memor$ or recall or think$) adj5 (declin$ or impair$ or domain$ or test$ or assess$ or function$ or batter$ or disorder$ or dysfunct$ or deficit$ or declin$ or abilit$ or problem$ or difficult$ or disturbance$ or disabilit$)).tw.

10. cognitive processes/ or cognition/ or awareness/ or cognitive reserve/ or executive function/ or learning/ or thinking/ or perception/ or memory/
11. exp psychological assessment/
12. MMSE.tw.
13. or/7-12

Neuroimaging

14. neuroimaging/
15. multi$modal imag$.tw.
16. ((compute$ or cerebral or CAT or CT or brain) adj5 (imag$ or scan$ or neuroimag$ or tomogra$ or marker$ or feature$ or x-ray)).tw.
17. magnetic resonance imaging/
18. (MRI or MRi or NMRI or NMRi).tw.
19. ((magn$ or resonance or MR or MTC or MT or NMR) adj5 (imag$ or scan$ or neuroimag$ or tomogra$ or marker$ or feature$)).tw.
20. or/14-19

Study type

21. prognosis/
22. observation methods/
23. randomized controlled trial.pt.
24. controlled clinical trial.pt.
25. exp cohort analysis/
26. (epidemiologic adj (study or studies)).ab,ti.
27. case control.ab,ti.
28. (cohort adj (study or studies)).ab,ti.
29. cohort analy$.ab,ti.
30. (follow up adj (study or studies)).ab,ti.
31. longitudinal.ab,ti.
32. retrospective$.ab,ti.
33. prospective$.ab,ti.
34. (observ$ adj3 (study or studies)).ab,ti.
35. or/21-34

Running the search

36. 6 and 13 and 20 and 35

Cochrane Central Register of controlled Trials (CENTRAL)

Stroke

1. MeSH descriptor: [Cerebrovascular Disorders] this term only
2. MeSH descriptor: [Basal Ganglia Cerebrovascular Disease] explode all trees
3. MeSH descriptor: [Brain Ischemia] this term only
4. MeSH descriptor: [Hypoxia-Ischemia, Brain] this term only
5. MeSH descriptor: [Ischemic Attack, Transient] this term only
6. MeSH descriptor: [Carotid Artery Diseases] this term only
7. MeSH descriptor: [Carotid Artery Thrombosis] this term only
8. MeSH descriptor: [Intracranial Arterial Diseases] this term only
9. MeSH descriptor: [Cerebral Arterial Diseases] this term only
10. MeSH descriptor: [Intracranial Embolism and Thrombosis] explode all trees
11. MeSH descriptor: [Intracranial Hemorrhages] explode all trees
12. MeSH descriptor: [Stroke] explode all trees
13. ((stroke* or apoplex* or cerebral vasc* or cerebrovasc* or cva or transient isch*mic attack* or tia))
14. ((brain or cerebr* or cerebell* or hemispher* or intracran* or intracerebral or infratentorial or supratentorial or space-occupying) NEAR/3 (isch*emi* or infarct* or thrombo* or emboli* or occlus* or hypoxi*))
15. (((brain or cerebr* or cerebell* or hemispher* or intracran* or intracerebral or infratentorial or supratentorial or intraventricular) NEAR/3 (h*morrhag* or h*matoma* or bleed*)))
16. #1 OR #2 OR #3 OR #4 OR #5 OR #6 OR #7 OR #8 OR #9 OR #10 OR #11 OR #12 OR #13 OR #14 OR #15

Dementia and cognitive impairment

17. MeSH descriptor: [Dementia] explode all trees
18. MeSH descriptor: [Memory Disorders] this term only
19. MeSH descriptor: [Neurocognitive Disorders] this term only
20. MeSH descriptor: [Cognition Disorders] this term only
21. MeSH descriptor: [Cognitive Dysfunction] this term only
22. (dement*)
23. (((attention or awareness or cognit* or neuropsych* or neurocognit* or neurobehav* or psycholog* or memor* or recall or think*) NEAR/3 (declin* or impair* or domain* or test* or assess* or function* or batter* or disorder* or dysfunct* or deficit* or declin* or abilit* or problem* or difficult* or disturbance* or disabilit*)))
24. MeSH descriptor: [Mental Processes] this term only
25. MeSH descriptor: [Cognition] this term only
26. MeSH descriptor: [Awareness] this term only
27. MeSH descriptor: [Cognitive Reserve] this term only
28. MeSH descriptor: [Executive Function] this term only
29. MeSH descriptor: [Learning] explode all trees
30. MeSH descriptor: [Thinking] this term only
31. MeSH descriptor: [Perception] this term only
32. MeSH descriptor: [Memory] this term only
33. MeSH descriptor: [Psychological Tests] explode all trees
34. (MMSE)
35. #17 OR #18 OR #19 OR #20 OR #21 OR #22 OR #23 OR #24 OR #25 OR #26 OR #27 OR #28 OR #29 OR #30 OR #31 OR #32 OR #33 OR #34

Neuroimaging

36. MeSH descriptor: [Diagnostic Imaging] this term only
37. MeSH descriptor: [Neuroimaging] this term only
38. MeSH descriptor: [Multimodal Imaging] this term only
39. MeSH descriptor: [Tomography, X-Ray Computed] this term only
40. (((compute* or cerebral or CAT or CT or brain) NEAR/3 (imag* or scan* or neuroimag* or tomogra* or marker* or feature* or x-ray)))
41. MeSH descriptor: [Magnetic Resonance Imaging] this term only
42. ((MRI or MRi or NMRI or NMRi))
43. (((magn* or resonance or MR or MTC or MT or NMR) NEAR/3 (imag* or scan* or neuroimag* or tomogra* or marker* or feature*)))
44. #36 OR #37 OR #38 OR #39 OR #40 OR #41 OR #42 OR #43

Running the search

45. #16 AND #35 AND #44

Supplement 2: Data extraction form

Study characteristics:

Year of stroke	Country	Setting	Followed-up/recruited (%)	Females (%)	Mean age Years (SD)	Stroke type	Excluded pre-stroke cognitive impairment or dementia	Excluded prior stroke	Proportion of population with CT imaging	When was the scan performed	Neuroimaging features reported	Who rated the CT scans

Cognitive follow-up:

Latest follow-up time	PSD N (%)	PSCI N (%)	Diagnostic criteria for PSD	Criteria for PSCI

Clinical and vascular risk factors of patients included in the latest follow-up:

Age	Females N (%)	Education	Hypertension (%)	Diabetes (%)	Hypercholesterolemia (%)	Atrial fibrillation (%)	Smoking (%)	Alcohol intake (%)	APOE status (%)	Ischaemic heart disease (%)	Prior stroke/TIA (%)	Stroke severity	Proportion with cognitive impairment prior to stroke (%)

Neuroimaging features:

			Unadjusted analysis					Adjusted analysis				
Neuroimaging feature	Developed PSD/PSCI	Not developed PSD/PSCI	Effect size	Sig. level	Statistical test	Associated with PSD	Associated with PSCI	Effect size	Sig. level	Statistical test	Associated with PSD	Associated with PSCI

Data for meta-analyses:

Risk factor	Definition	Biomarker present & developed PSD/PSCI	Total that developed PSD/PSCI	Biomarker present & did not develop PSD/PSCI	Total that did not develop PSD/PSCI	OR presented in study
Female						
Low education						
Hypertension						
Diabetes						
Hypercholesterolemia						
Atrial fibrillation						
Smoking						
High alcohol intake						
APOE						
Ischaemic heart disease						
Prior stroke						
Prior TIA						
Pathological stroke type						
Presence of atrophy						
Presence of WML						
Presence of pre-existing stroke lesions						

Supplement 3: Study characteristics of included post-stroke dementia and post-stroke cognitive impairment studies

28 studies[1-28] (described in 41 papers)[29-41] were eligible for inclusion.

Study	Year of stroke	Country	Setting	Followed-up / Recruited (%)	Females included in follow-up (%)	Mean age years (SD)	Stroke type	Excluded pre-stroke cognitive impairment or dementia?	Excluded prior stroke?	Neuroimaging features reported	Performed adjusted analyses
Loeb et al,[14] 1992	1979-1984	Italy	Inpatient	108/108 (100%)	19/108 (18%)	65.1 (9.5)	Lacunar infarct	Dementia	NS	Acute stroke features, atrophy, WML	No
Kase et al, [11] 1998	1982-1994	United States	Inpatient & outpatient	74/251 (29%)	40/74 (54%)	78.6 (0.8)	Isch & ICH	Dementia	No	Acute stroke features, pre-existing stroke lesions, WML	No
Miyao et al,[17] 1992	1984-1990	Japan	Inpatient	215/215 (100%)	76/215 (35%)	NS	Lacunar infarct	Dementia	Yes	WML*	No
House et al,[9] 1990	1985-1986	England	Stroke register	115/NS (NS)	NS/115	NS	Isch & ICH	No	Yes	Acute stroke features, atrophy, WML	No
Bornstein et al,[6] 1996	1988 - 1990	Israel	Inpatient	157/199 (79%)	71/157 (45%)	72.3 (6.8)	Isch	Cognitive impairment	Yes	Pre-existing stroke lesions*	No
Andersen et al,[2] 1996	1991-1992	Denmark	Inpatient & outpatient	127/188 (68%)	NS/127	NS	Isch & ICH	Dementia	No	Acute stroke features, atrophy	Yes
Barba et al,[4] 2000	1994-1995	Spain	Inpatient	251/327 (77%)	117/251 (47%)	69 (13)	Isch & ICH	'Previous long lasting mental retardation'	No	Acute stroke features, pathological stroke type*	Yes
Mackowiak-Cordoliani et al,[15] 2003	1995-1996	France	Inpatient	144/171 (84%)	78/144 (54%)	NS	Isch & ICH	Dementia	No	Acute stroke features, atrophy, pathological stroke type*, pre-existing stroke lesions*, WML	Yes
Gomez-Vierra et al,[8] 2002	1997-2000	Cuba	Inpatient	301/401 (75%)	133/301 (44%)	NS	Isch	Dementia	No	Acute stroke features, atrophy, pre-existing stroke lesions	Yes
Chausson et al,[7] 2010	1998-1999	Martinique	Inpatient & outpatient & primary care	229/580 (39%)	NS/229	NS	Isch & ICH	No	No	Acute stroke features, WML	No

Study	Year of stroke	Country	Setting	Followed-up / Recruited (%)	Females included in follow-up (%)	Mean age years (SD)	Stroke type	Excluded pre-stroke cognitive impairment or dementia?	Excluded prior stroke?	Neuroimaging features reported	Performed adjusted analyses
Klimkowicz-Mrowiec et al,[12] 2006	2000-2001	Poland	Inpatient	195/250 (78%)	83/195 (43%)	NS	Isch & ICH	Dementia	No	Acute stroke features, atrophy*, pathological stroke type*, pre-existing stroke lesions, WML*	Yes
Rasquin et al,[21] 2004	2000-2001	Netherlands	Inpatient & outpatient	144/176 (82%)	NS/144	NS	Isch	Dementia, MMSE greater than or equal to 15	Yes	Acute stroke features, atrophy*, pre-existing stroke lesions*, WML*	Yes
Appleton et al,[3] 2020	2001 -2013	23 countries	Inpatient	1930-2269/4011 (~50%)	NS	NS	Isch & ICH	No	No	Acute stroke features, atrophy, pre-existing stroke lesions, WML	Yes
Thein et al,[27] 2007	2004-2004	Malaysia	Inpatient	60/60 (100%)	21/60 (35%)	63.17 (10.53)	Isch	No	Yes	WML	No
Sundar et al,[25] 2010	2004-2006	India	Inpatient	164/NS	61/164 (37%)	63 (NS)	Isch	Cognitive impairment	Yes	Acute stroke features	No
Moulin et al,[18] 2016	2004-2009	France	Inpatient	218/264 (83%)	100/218 (46%)	NS	ICH	Cognitive impairment	No	Acute stroke features, atrophy*, WML*	Yes
Alexandrova et al,[1] 2016	2006-2009	Bulgaria	Inpatient	47/63 (75%)	21/47 (45%)	NS	Isch	Dementia and 'neurological or psychiatric disorders that can cause cognitive dysfunction'	No	Acute stroke features	No
Biffi et al,[5] 2016	2006-2013	United States	Inpatient	435/738	209/435 (48%)	71.2 (11.3)	ICH	Dementia and dementia within 6 months of stroke	No	Acute stroke features, WML	Yes
Zhang et al, [28] 2012	2009-2010	China	Inpatient	577/633 (91%)	183/577 (32%)	NS	Isch & ICH	Dementia and cognitive impairment	Yes	Acute stroke features, pathological stroke type*, WML	Yes
Saini et al,[23] 2014	2009-2012	Singapore	Inpatient	318/400 (80%)	96/318 (30%)	59.8 (11.4)	Isch & TIA	Dementia or >3.38 on the baseline IQCODE	No	Acute stroke features, atrophy, pre-existing stroke lesions, WML	Yes

Study	Year of stroke	Country	Setting	Followed-up / Recruited (%)	Females included in follow-up (%)	Mean age years (SD)	Stroke type	Excluded pre-stroke cognitive impairment or dementia?	Excluded prior stroke?	Neuroimaging features reported	Performed adjusted analyses
Jacquin et al,[10] 2014	2010-2012	France	Inpatient	220/280 (79%)	97/220 (44%)	66.1 (16.6)	Isch & ICH	Dementia & severe cognitive impairment	Yes	Acute stroke features, pathological stroke type*, pre-existing stroke lesions, WML	Yes
Pinzon et al,[19] 2018	2017	Indonesia	Medical record & registry	110/110 (100%) - retrospective study	38/110 (35%)	NS	Isch	Dementia	No	Acute stroke features	Yes
Prodjohardjono et al,[20] 2020	2018-2019	Indonesia	Inpatient	56/83 (67%)	21/56 (38%)	NS	Isch	Dementia and mild cognitive impairment	Yes	Acute stroke features	Yes
Lin et al,[13] 1998	NS	China	NS	100/NS (NS)	36/100 (36%)	NS	Isch	Dementia	No	Acute stroke features, atrophy*, WML*	Yes
Mehrabian et al,[16] 2015	NS	Bulgaria	Inpatient	74/85 (87%)	NS/74	NS	Isch	Cognitive impairment	yes	Acute stroke features, atrophy, WML	No
Renjen et al[22], 2015	NS	India	Inpatient & outpatient	50/NS (NS)	18/50 (36%)	61.82 (NS)	Isch & ICH	No	No	Acute stroke features, atrophy, pathological stroke type*, pre-existing stroke lesions, WML	No
Schmidt et al,[25] 2015	NS	United States	Outpatient	41/45 (91%)	16/41 (39%)	NS	Supratentorial infarct	Dementia and cognitive impairment	Yes	Acute stroke features, atrophy, WML	Yes
Tang et al[26], 2004	NS	Hong Kong	Inpatient	279/484 (58%)	127/279 (46%)	70.9 (9.6)	Isch & ICH	No	No	Acute stroke features, atrophy, pathological stroke type*, WML*	Yes

**neuroimaging feature included in meta-analysis*
Performed adjusted analysis = performed adjusted analysis within the study (i.e. may not have included the imaging feature in the multivariable model if it did not reach statistical significance following univariate analysis).
23 countries = Australia, Canada, China, Denmark, Egypt, Eire, Georgia, Greece, Hong Kong, India, Italy, Norway, Malaysia, New Zealand, Philippines, Poland, Romania, Singapore, Spain, Sri Lanka, Sweden, Turkey, UK.
***Abbreviations:** Isch, ischaemic stroke; ICH, intracerebral haemorrhage; WML, white matter lesions; NS, not stated; MMSE, mini-mental state examination; IQCODE, Informant Questionnaire on Cognitive Decline in the Elderly; PSCI, post-stroke cognitive impairment; PSD, post-stroke dementia; SD, standard deviation.*

Supplement 4: Clinical risk factors of stroke population included in cognitive follow-up

Author	Age	Females	Education	Hypertension	Diabetes	Hypercholesterolemia	Atrial fibrillation	Smoking	Alcohol	APOE	IHD	Prior stroke/ TIA	Stroke severity	Pre-stroke cognitive impairment/dementia
Alexandrova et al,[1] 2016	Median=6, Range=56-76	45%	8th grade= 19%, High school= 51%, College 11%, University= 19%	96%	28%	NS	N/A	40%	Alcohol abuse=21 %	N/A	N/A	History of stroke=21%	NIHSS Median= 5, Range= 4-6	Excluded patients with pre-stroke dementia and 'neurological or psychiatric disorders that can cause cognitive dysfunction'
Andersen et al,[2] 1996	NS	NS	NS	NS	NS	N/A	NS	NS	N/A	N/A	NS	N/A	N/A	Excluded patients with pre-stroke dementia.
Appleton et al,[3] 2020	NS	NS	N/A	NS	NS	NS	NS	NS	NS	N/A	NS	NS	NS	Did not exclude or assess pre-stroke cognitive impairment/dementia.
Barba et al,[4] 2000	Mean=69, SD=13	47%	Illiterate=27%, Able to read and write=52%, Below high school=14%, High school=4%, University=1 % (Not reported in the study=2%)	60%	25%	N/A	13%	47%	42%	N/A	Myocardial infarction=12%, Heart failure=6%, Mitral valve disease=7%, Aortic valve disease=4%, Aortic arch calcification=27%, Intermittent claudication=8%	Prior stroke=12%, Prior TIA=14%	Canadian Neurological Scale mean=7	SS-IQCODE mean score (SD): PSD group=62 (12.4) No PSD group=53 (3.2)<br><br>25% of patients who were followed-up had pre-stroke dementia.
Biffi et al,[5] 2016	Mean=71, SD=11.3	47%	Less than 10 years=49%, More than or equal to 10 years=51%	70%	15%	N/A	9%	N/A	N/A	Minor allele frequency APOE E2=0.08, Minor allele frequency APOE E4=0.20	Ischaemic heart disease=15%	Prior lobar ICH =3%, Prior non lobar ICH=0.2%, Prior ischaemic stroke or TIA=5%	N/A	Excluded patients with pre-stroke dementia and dementia at 6 months post-stroke.
Bornstein et al,[6] 1996	Mean=72, SD=6.8	45%	N/A	NS	NS	NS	N/A	N/A	N/A	N/A	NS	Excluded previous cerebral insults	N/A	Excluded patients reported to have pre-stroke cognitive impairment.

Author	Age	Females	Education	Hypertension	Diabetes	Hypercholesterolemia	Atrial fibrillation	Smoking	Alcohol	APOE	IHD	Prior stroke/ TIA	Stroke severity	Pre-stroke cognitive impairment/dementia
Chausson et al,[7] 2010	NS	NS	N/A	NS	NS	NS	NS	NS	N/A	N/A	N/A	N/A	N/A	Did not exclude or assess pre-stroke cognitive impairment/dementia.
Gomez-Vierra et al,[8] 2002	NS	44%	Up to primary school= 66%, Beyond primary school= 34%	76%	27%	54%	10%	50%	34%	N/A	9%	Prior stroke=18%, Prior TIA=27%	N/A	Excluded patients with pre-stroke dementia.
House et al,[9] 1990	NS	NS	N/A	NS	N/A	N/A	N/A	N/A	N/A	N/A	NS	Excluded prior stroke	N/A	Did not exclude or assess pre-stroke cognitive impairment/dementia.
Jacquin et al,[10] 2014	Mean=66, SD=16.6	44%	Primary=47%, Secondary=37%, Higher=15%	56%	15%	36%	14%	42%	4%	N/A	Myocardial infarction=5%	Excluded prior stroke, Prior TIA=8%	NIHSS Median= 3, IQR=6	Excluded patients with pre-stroke cognitive impairment and pre-stroke dementia.
Kase et al,[11] 1998	76.2	54%	<8th grade=11% (out of 72), <High school=21% (out of 72), High school degree=42% (out of 72), >High school degree=26% (out of 72)	NS	NS	N/A	NS	NS	N/A	N/A	N/A	N/A	NS	Excluded patients with pre-stroke dementia.
Klimkowicz-Mrowiec et al,[12] 2006	NS	43%	Elementary=81, High school & university=114	75%	22%	NS	17%	33%	N/A	N/A	Ischaemic heart disease=54%, Myocardial infarction=14%	Prior stroke=17%, Prior TIA=7%	N/A	Excluded patients with pre-stroke dementia.
Lin et al,[13] 1998	NS	36%	NS	N/A	N/A	N/A	N/A	N/A	N/A	N/A	N/A	NS	NS	Excluded patients with pre-stroke dementia.

Author	Age	Females	Education	Hypertension	Diabetes	Hypercholesterolemia	Atrial fibrillation	Smoking	Alcohol	APOE	IHD	Prior stroke/ TIA	Stroke severity	Pre-stroke cognitive impairment/dementia
Loeb et al,[14] 1992	Mean=65.1 SD=9.5	18%	Average years of education=8	NS	NS	NS	N/A	NS	N/A	N/A	NS	N/A	N/A	Excluded pre-stroke dementia.
Mackowiak-Cordoliani et al,[15] 2003	Median =72	54%	Achieved primary educational level=81%	54%	11%	25%	NS	21%	23%	N/A	High risk cardiopathy=30%, Stenosis of cervical artery >50%=21%, Cervical artery atheroma=14%, Cardioembolism=26%	Prior stroke=13%, Prior TIA=17%	N/A	Excluded patients with pre-stroke dementia. 61% who were included were assessed as having pre-stroke cognitive impairment.
Mehrabian et al,[16] 2015	NS	NS	NS	NS	NS	N/A	N/A	N/A	NS	N/A	N/A	N/A	NS	Excluded pre-stroke cognitive impairment.
Miyao et al,[17] 1992	LA+=71.3, LA-=65.5	LA+=37, LA-=39	N/A	LA +=62, LA-=66	LA +=14, LA- =11	LA+=7, LA-=16	LA +=7, LA-=1	N/A	N/A	N/A	LA+=6, LA-=3	Excluded prior stroke	N/A	Excluded pre-stroke dementia.
Moulin et al,[18] 2016	Median=67.5, IQR=55-76	46%	Less than or equal to 8 years=60%, More than 8 years=40%	65%	14%	32%	N/A	19%	30%	N/A	10%	Prior stroke or TIA=15%	NIHSS score: PSD group=10 (4-16), No PSD group=7 (3-16)	Excluded pre-stroke dementia. 13% of patients included in follow-up had pre-stroke cognitive impairment.
Pinzon et al,[19] 2018	40-50 years=11%, 51-60 years =33%, 61-70 years =31%, >70 years =26%	35%	≤12 years=60%, >12 years=40%	70%	41%	51%	N/A	44%	N/A	N/A	N/A	Prior stroke= 42%	N/A	Excluded pre-stroke dementia

Author	Age	Females	Education	Hypertension	Diabetes	Hypercholesterolemia	Atrial fibrillation	Smoking	Alcohol	APOE	IHD	Prior stroke/ TIA	Stroke severity	Pre-stroke cognitive impairment/dementia
Prodjohardjono et al,[20] 2020	PSCI: Mean=61.63 SD=8.47, No PSCI: Mean=58.67 SD =9.01	38%	PSCI: Median= 12 Range=4-16, Non-PSCI: Median= 12 Range=6-22	NS	NS	Anti-dyslipidemic use= 77%	N/A	NS	N/A	N/A	N/A	Excluded prior stroke	NIHSS minor= 50%, NIHSS moderate = 50%	Excluded mild cognitive impairment and pre-stroke dementia
Rasquin et al,[21] 2004	NS	NS	NS	NS	NS	NS	N/A	NS	N/A	N/A	NS	Excluded prior stroke	N/A	Excluded pre-stroke dementia or MMSE greater than or equal to 15
Renjen et al,[22] 2015	Mean=61.82	36%	Studied up to grade 10 or more=80%	N/A	N/A	N/A	N/A	N/A	N/A	N/A	N/A	Recurrent stroke during follow-up=10%	PSD group: median NIHSS=6, No PSD group: NS	2% had pre-stroke cognitive impairment no dementia
Saini et al,[23] 2014	Mean=59.8 SD=11.4	30%	Mean years of education=7.7, SD=4.3	71%	40%	77%	11%	41%	N/A	N/A	Ischaemic heart disease=21%	Prior stroke/TIA=19%	NS	Excluded pre-stroke dementia or >3.38 on the baseline IQCODE
Schmidt et al,[24] 1993	Range=33-82	39%	NS	59%	27%	37%	N/A	24%	N/A	N/A	Cardiac disease=71%	N/A	N/A	Excluded pre-stroke cognitive impairment or dementia
Sundar et al,[25] 2010	Mean=63, Range 52-81	37%	Studied up to 5th grade at least=93%	51%	23%	34%	N/A	46%	N/A	N/A	N/A	Excluded prior stroke	N/A	Excluded patients with pre-stroke cognitive impairment
Tang et al,[26] 2004	Mean=70.9 SD=9.6	46%	Mean years=4.3, SD=4.2	67%	29%	NS	5%	46%	N/A	N/A	Ischemic heart disease=9%	Prior stroke=28%, Prior TIA=5%	NIHSS mean=6.7, SD=5.6	Pre-stroke dementia=8%
Thein et al,[27] 2007	Mean=63.17, SD=10.53	35%	N/A	NS	NS	NS	N/A	NS	NS	N/A	NS	Excluded prior stroke	NS	N/A

Author	Age	Females	Education	Hypertension	Diabetes	Hypercholesterolemia	Atrial fibrillation	Smoking	Alcohol	APOE	IHD	Prior stroke/ TIA	Stroke severity	Pre-stroke cognitive impairment/dementia
Zhang et al,[28] 2012	Greater than or equal to 65 years= 43%	32%	Less than 7 years education= 29%, More than 7 years education= 71%	70%	27%	33%	4%	N/A	N/A	N/A	N/A	Excluded prior stroke and TIA	NIHSS at admission equal to or more than 6=21%	Excluded pre-stroke cognitive impairment and dementia

Abbreviation: APOE, apolipoprotein E; ICH, intracerebral haemorrhage; LA+, leukoaraiosis positive; LA-, leukoaraiosis; MMSE, Mini Mental State Examination; N/A, not applicable; NIHSS, NIH stroke scale; NS, not stated; PSCI, post-stroke cognitive impairment; PSD, post-stroke dementia; SS-IQCODE, Shortened Spanish version of the Informant Questionnaire on Cognitive Decline in the Elderly; TIA, transient ischaemic attack

Supplement 5: Cognitive follow-up of PSD and PSCI studies

Study	PSD N (%)	Criteria for PSD	PSCI N (%)	Criteria for PSCI	Latest follow-up time
Barba et al,[4] 2000	75/251 (30%)	DSM-IV	N/A	N/A	3 months
Biffi et al,[5] 2016	139/435 (32%)	ICD-9 codes in medical records and/or TICS-m>20	N/A	N/A	Median=47.4 months, IQR=43.4-51.2 months
Bornstein et al,[6] 1996	56/157 (36%)	DSM-III-R	N/A	N/A	5 years or until dementia diagnosis
Klimkowicz-Mrowiec et al,[12] 2006	44/195 (23%)	DSM-IV or IQCODE≥104	N/A	N/A	3 months
Lin et al,[13] 1998	50/100 (50%)	NINDS-AIREN and CDR≥1	N/A	N/A	3-4 months
Loeb et al,[14] 1992	25/108 (23%)	DSM-III-R and MMSE<24	N/A	N/A	4 years (mean 55.8 months)
Mackowiak-Cordoliani et al,[15] 2003	34/144 (24%)	ICD-10	N/A	N/A	Within 3 years (included patients who had died during follow-up)
Miyao et al,[17] 1992	24/215 (11%)	DSM-III-R and MMSE <20	N/A	N/A	At least once a month until end of study or death (average 25-29 months)
Moulin et al,[18] 2016	63/218 (29%)	NIA-AA	N/A	N/A	Median=6 years
Tang et al,[26] 2004	55/279 (20%)	DSM-IV	N/A	N/A	3 months
Rasquin et al,[21] 2004	14/144 (10%)	DSM-IV	85/144 (59%)	Neuropsychological battery (at least one cognitive deficit)	12 months
Alexandrova et al,[1] 2016	N/A	N/A	20/47 (43%)	MMSE<24	12 months
Andersen et al,[2] 1996	N/A	N/A	33/127 (26%)	MDRS≤127	12 months
Appleton et al,[3] 2020	N/A	N/A	NS	MMSE-M, TICS-M, verbal fluency	3 months
Chausson et al,[7] 2010	N/A	N/A	18/229 (8%)	MMSE <11	5 years
Gomez-Vierra et al,[8] 2002	N/A	N/A	88/301 (29%)	Neuropsychological battery and MMSE	6 months
House et al,[9] 1990	N/A	N/A	24/115 (21%)	MMSE≤23	12 months
Jacquin et al,[10] 2014	N/A	N/A	104/220 (47%)	MMSE≤26 and MoCA≤26 or neuropsychological battery. Dementia diagnosed according to DSM-IV.	3 months
Kase et al,[11] 1998	N/A	N/A	NS	MMSE<24, neuropsychological battery, dementia diagnosed according to DSM-III	6 months
Mehrabian et al,[16] 2015	N/A	N/A	59/74 (80%)	Winblad et al. criteria (MCI) and impairment in one or more cognitive domains. Dementia diagnosed according to DSM-IV and NINDS-AIREN.	12 months
Pinzon et al,[19] 2018	N/A	N/A	75/110 (68%)	MoCA-INA<26 and clock drawing test>1	3-6 months
Prodjohardjono et al,[20] 2020	N/A	N/A	35/56 (63%)	MoCA-INA<26	3 months

Study	PSD N (%)	Criteria for PSD	PSCI N (%)	Criteria for PSCI	Latest follow-up time
Renjen et al,[22] 2015	N/A	N/A	36/50 (72%)	PGI-BBD≥18, DSM used to diagnose dementia	12 months
Saini et al,[23] 2014	N/A	N/A	76/318 (24%)	Neuropsychological battery (impaired in 1 or more domains). Dementia diagnosed according to DSM-IV.	3-6 months
Schmidt et al,[24] 1993	N/A	N/A	15/41 (37%)	MDRS<137	6 months
Sundar et al,[25] 2010	N/A	N/A	52 (32%)	MMSE less than 24 and/or frontal assessment battery	3 months
Thein et al,[27] 2007	N/A	N/A	24/60 (40%)	MMSE score <24	3 months
Zhang et al,[28] 2012	N/A	N/A	177 (31%)	Neuropsychological battery (impaired in 1 or more domains)	3 months

Abbreviations: DSM-IV, Diagnostic and Statistical Manual of Mental Disorders – fourth edition; ICD-10, International Classification of Diseases – Tenth Revision; ICD-9, *International Classification of Diseases – Ninth Revision; IQCODE, Informant Questionnaire on Cognitive Decline in the Elderly; IQR, Inter-quartile range; DSM-III-R, Diagnostic and Statistical Manual of Mental Disorders – Third Edition Revised; MDRS, Mattis Dementia Rating Scale; MMSE, mini-mental state examination; MoCA, Montreal Cognitive Assessment; MoCA-Ina, Montreal Cognitive Assessment – Indonesian version; N/A, not applicable; NIA-AA, National Institute on Aging and Alzheimer's association; NINDS-AIREN, National Institute of Neurological disorders and Stroke and the Association Internationale pour la Recherche et l'Enseignement en Neurosciences; NS, not specified; PGI-BBD, PGI Battery of Brain Dysfunction; PSCI, post-stroke cognitive impairment; PSD, post-stroke dementia; TICS-M, Modified Telephone Interview for Cognitive Status.*

Supplement 6: Atrophy

eTable 6.1: PRESENCE of cerebral atrophy and post-stroke DEMENTIA

				Unadjusted analysis				Adjusted analysis			
**Study**	**Neuroimaging feature**	**Developed PSD**	**Not developed PSD**	**Effect size**	**Sig. level**	**Statistical test**	**Associated with PSD**	**Effect size**	**Sig. level**	**Statistical test**	**Associated with PSD**
Klimkowicz-Mrowiec et al,[12] 2006	Cerebral atrophy	Present=79%, Absent=21%	Present=73%, Absent=27%	OR=1.41, CI=0.62-3.19	0.412	Logistic regression (calculated by review authors)	No*	N/A	N/A	N/A	N/A
Lin et al,[13] 1998	Cerebral atrophy	Present=92%, Absent=8%	Present=84%, Absent=16%	OR=2.19, CI=0.61-7.81	0.227	Logistic regression (calculated by review authors)	No*	N/A	N/A	N/A	N/A
Moulin et al,[18] 2016	Cortical atrophy	Present=95%, Absent=5%	Present=77%, Absent=23% (N=5 missing from study's analysis)	OR=5.86, CI=1.73-19.87	0.005	Logistic regression (calculated by review authors)	Yes*	N/A	N/A	N/A	N/A
Rasquin et al,[21] 2004	Brain atrophy	NS	NS	OR=7.7, CI=0.9-65.2	0.06	Chi-square or Fischer's exact test	No*	N/A	N/A	N/A	N/A

*finding visualised in the harvest plot (Figure 2)
Abbreviations: N/A, not applicable, NS, not stated

eTable 6.2: PRESENCE of cerebral atrophy and post-stroke COGNITIVE IMPAIRMENT

				Unadjusted analysis				Adjusted analysis			
**Study**	**Neuroimaging feature**	**Developed PSCI**	**Not developed PSCI**	**Effect size**	**Sig. level**	**Statistical test**	**Associated with PSCI**	**Effect size**	**Sig. level**	**Statistical test**	**Associated with PSCI**
Andersen et al,[2] 1996	Central atrophy	N/A	N/A	N/A	N/A	N/A	N/A	NS	<0.001	Stepwise multiple regression	Yes*
Gomez-Vierra et al,[8] 2002	Cerebral atrophy	Present=56%, Absent=44%	Present=55%, Absent=45%	RR=1.02, CI=0.72-1.46	0.9	Chi-square test	No*	N/A	N/A	N/A	N/A
Rasquin et al,[21] 2004	Brain atrophy	NS	NS	OR=2.2, CI=0.9-5.1	0.06	Chi-square or Fischer's exact test	No*	N/A	N/A	N/A	N/A
Renjen et al,[22] 2015	Cortical atrophy	'Cognitive impairment was more frequent in patients with… cortical atrophy (100% in severe atrophy)'	NS	NS	0.337	Student's t-test, Mann-Whitney U test, Fisher's exact test, and Chi-square test	No*	N/A	N/A	N/A	N/A
Saini et al,[23] 2014	Significant global cortical atrophy	N/A	N/A	N/A	N/A	N/A	N/A	OR=3.07, CI=1.48-6.40	<.01	Logistic regression	Yes*
Schmidt et al,[24] 1993	Moderately severe ventricular atrophy	46.6%	No cognitive impairment: 12.5%, Transient cognitive impairment: 10%	NS	<0.05	Fisher's exact test	Yes*	NS	NS	Stepwise backward regression	No*

*finding visualised in the harvest plot (Figure 2)
Abbreviations: N/A, not applicable, NS, not stated

eTable 6.3 SEVERITY of cerebral atrophy and post-stroke DEMENTIA

				Unadjusted analysis				Adjusted analysis			
**Study**	**Neuroimaging feature**	**Developed PSD**	**Not developed PSD**	**Effect size**	**Sig. level**	**Statistical test**	**Associated with PSD**	**Effect size**	**Sig. level**	**Statistical test**	**Associated with PSD**
Mackowiak-Cordoliani et al,[15] 2003	Cerebral atrophy score	Median=2, range 0-3	Median=1, Range=0-3	RR=2.19, CI=1.5-3.17	<0.05	Kaplan-Meier survival analysis using the log rank test	Yes*	N/A	N/A	N/A	N/A
Klimkowicz-Mrowiec et al,[12] 2006	Cerebral atrophy (0:none, 1:mild, 2:moderate, 3:severe)	0=21%, 1=40%, 2=19%, 3=21% (N=1 missing from the study's calculation)	0=27%, 1=52%, 2=15%, 3=6%	NS	0.01	Chi-square	Yes*	NS	NS	Backward stepwise logistic regression	No*
Lin et al,[13] 1998	Cerebral atrophy (0: none, 1: mild, 2: moderate, 3: severe)	0=8%, 1=46%, 2=38%, 3=8%	0=16%, 1=66%, 2=16%, 3=2%	NS	<0.05	Exact 2 x k table test for trend	Yes*	N/A	N/A	N/A	N/A
Loeb et al,[14] 1992	Cerebral atrophy	NS	NS	NS	<0.06	t-test	No*	N/A	N/A	N/A	N/A
Tang et al,[26] 2004	Cerebral atrophy index	Mean=10.51, SD=4.10	Mean=7.73, SD=3.28	OR=1.229, CI=1.128-1.338	<0.001	Univariate logistic regression	Yes*	OR=1.148, CI=1.015-1.297	<0.027	Multivariate logistic regression	Yes*
Moulin et al,[18] 2016	Cortical atrophy (0: none, 1: mild, 2: moderate, 3: severe)	0=5%, 1=22%, 2=51%, 3=22%	0=22%, 1=36%, 2=32%, 3=7% (N=5 missing from study's analysis)	NS	NS	NS	NS	0:reference 1: SHR=1.3 (0.34-5.07), 2: SHR=2.35 (0.6-9.770, 3: SHR=3.15 (0.69-14.35)	0:reference 1: p=0.7, 2: p=0.22 3: p=0.14	Backward stepwise multivariate analysis	No*

*finding visualised in the harvest plot (Figure 2)
Abbreviations: N/A, not applicable, NS, not stated

eTable 6.4: SEVERITY of cerebral atrophy and post-stroke COGNITIVE IMPAIRMENT

				Unadjusted analysis				Adjusted analysis			
**Study**	**Neuroimaging feature**	**Developed PSCI**	**Not developed PSCI**	**Effect size**	**Sig. level**	**Statistical test**	**Associated with PSCI**	**Effect size**	**Sig. level**	**Statistical test**	**Associated with PSCI**
Appleton et al,[3] 2020	Cerebral atrophy score (of 4), median (IQR)	N/A	N/A	N/A	N/A	N/A	N/A	Refer to study for each cognitive measure	Refer to study for each cognitive measure	Multiple linear regression	Associated with t-MMSE, TICS-M, verbal fluency*
Gomez-Vierra et al,[8] 2002	Intensity of cerebral atrophy	light=18%, moderate/intense=38%	light=31%, moderate/intense=33%	RR=0.79, CI=0.48-1.32	0.36	Chi-square test	No*	N/A	N/A	N/A	N/A
Schmidt et al,[24] 1993	Cortical atrophy	NS	NS	NS	NS	Fischer's Exact test	No*	N/A	N/A	N/A	N/A

*finding visualised in the harvest plot (Figure 2)
Abbreviations: N/A, not applicable, NS, not stated

eTable 6.5: PRESENCE of LOCALISED atrophy and post-stroke DEMENTIA

				Unadjusted analysis				Adjusted analysis			
**Study**	**Neuroimaging feature**	**Developed PSD**	**Not developed PSD**	**Effect size**	**Sig. level**	**Statistical test**	**Associated with PSD**	**Effect size**	**Sig. level**	**Statistical test**	**Associated with PSD**
Mackowiak-Cordoliani et al,[15] 2003	Medial temporal lobe atrophy	NS	NS	RR=2.3, CI=1.1-4.7	.02	Kaplan-Meier survival analysis using the log rank test	Yes*	NS	>0.05	Cox proportional hazards regression analysis	No

*finding visualised in the harvest plot (Figure 2)
Abbreviations: N/A, not applicable, NS, not stated

eTable 6.6: PRESENCE of LOCALISED atrophy and post-stroke COGNITIVE IMPAIRMENT

				Unadjusted analysis				Adjusted analysis			
**Study**	**Neuroimaging feature**	**Developed PSCI**	**Not developed PSCI**	**Effect size**	**Sig. level**	**Statistical test**	**Associated with PSCI**	**Effect size**	**Sig. level**	**Statistical test**	**Associated with PSCI**
Mehrabian et al,[16] 2015	Hippocampal atrophy	N/A	N/A	Refer to study paper for each neuropsychological measure	Refer to study paper for each neuropsychological measure	Simultaneous multiple regression of neuropsychological measures	Yes – significant association with all neuropsychological measures*	N/A	N/A	N/A	N/A

*finding visualised in the harvest plot (Figure 2)
Abbreviations: N/A, not applicable, NS, not stated

eTable 6.7: MISCELLANEOUS atrophy and post-stroke COGNITIVE IMPAIRMENT

				Unadjusted analysis				Adjusted analysis			
**Study**	**Neuroimaging feature**	**Developed PSCI**	**Not developed PSCI**	**Effect size**	**Sig. level**	**Statistical test**	**Associated with PSCI**	**Effect size**	**Sig. level**	**Statistical test**	**Associated with PSCI**
Schmidt et al,[24] 1993	Indices of ventricular atrophy: frontal horn ratio (%)	Mean=35.9, SD=5.6	No cognitive impairment: Mean=32.6, SD=5.6; Transient cognitive impairment: Mean=33.9, SD=4.9	NS	NS	Fischer's Exact test	No	N/A	N/A	N/A	N/A
Schmidt et al,[24] 1993	Indices of ventricular atrophy: third ventricular ratio (%)	Mean=4.6, SD=1.6	No cognitive impairment: Mean=3.1, SD=1.5, Transient cognitive impairment: Mean=3.2, SD=1.4	NS	NS	Fischer's Exact test.	Yes	N/A	N/A	N/A	N/A
Schmidt et al,[24] 1993	Indices of ventricular atrophy: ventricle to intracranial cavity ratio (%)	Mean=7.6, SD=3.1	No cognitive impairment: Mean=5.4, SD=2.2, Transient cognitive impairment: Mean=5.9, SD=1.4	NS	NS	Fischer's Exact test.	Yes	N/A	N/A	N/A	N/A
Schmidt et al,[24] 1993	Measures of cortical atrophy: sylvian fissure width (mm)	Mean=5.1, SD=2.5	No cognitive impairment: Mean=3.2, SD=1.7, Transient cognitive impairment: Mean=3.9, SD=2.4	NS	NS	Fischer's Exact test	Yes	N/A	N/A	N/A	N/A
Schmidt et al,[24] 1993	Measures of cortical atrophy: frontal interhemispheric fissure ratio (%)	Mean=4.5, SD=1.7	No cognitive impairment: Mean=3.9, SD=1.3, Transient cognitive impairment: Mean=3.0, SD=1.6	NS	NS	Fischer's Exact test	No	N/A	N/A	N/A	N/A
Schmidt et al,[24] 1993	Measures of cortical atrophy: cortical sulci ratio (%)	Mean=13.2, SD=3.3	No cognitive impairment: Mean=10.4, SD=4.6, Transient cognitive impairment: Mean=12.4, SD=4.6	NS	NS	Fischer's Exact test	Yes	N/A	N/A	N/A	N/A
Gomez-Vierra et al,[8] 2002	Cerebral atrophy	Localised=19%, Generalised=36%	Localised=23%, Generalised=31%	RR=0.78, CI=0.48-1.29	0.33	Chi-square test	No	N/A	N/A	N/A	N/A

*finding visualised in the harvest plot (Figure 2)
Abbreviations: N/A, not applicable, NS, not stated

Supplement 7: White matter lesions

eTable 7.1: PRESENCE of WML and post-stroke DEMENTIA

				Unadjusted analysis				Adjusted analysis			
**Study**	**Neuroimaging feature**	**Developed PSD**	**Not developed PSD**	**Effect size**	**Sig. level**	**Statistical test**	**Associated with PSD**	**Effect size**	**Sig. level**	**Statistical test**	**Associated with PSD**
Klimkowicz-Mrowiec et al,[12] 2006	Leukoaraiosis	Present=46%, Absent=54%	Present=27%, Absent=73%	NS	0.02	Chi-square	Yes*	NS	NS	Backward stepwise logistic regression	No*
Lin et al,[13] 1998	Subcortical WML	Present=94%, Absent=4% (N=1 missing from study's analysis)	Present=90%, Absent=10%	OR=2.61, CI=0.48-14.15	0.2657	Logistic regression (calculated by review authors)	No	N/A	N/A	N/A	N/A
Lin et al,[13] 1998	Overall WML	Present=96%, Absent=4%	Present=92%, Absent=8%	OR=2.09, CI=0.36-11.95	0.4086	Logistic regression (calculated by review authors)	No*	N/A	N/A	N/A	N/A
Lin et al,[13] 1998	Periventricular changes	Present=88%, Absent=12%	Present=92%, Absent=8%	CI=0.64, CI=0.17-2.41	0.5076	Logistic regression (calculated by review authors)	No	N/A	N/A	N/A	N/A
Miyao et al,[17] 1992	Leukoaraiosis	Present=88%, Absent=13%	Present=39%, Absent=61%	NS	<0.001	Chi-square	Yes*	N/A	N/A	N/A	N/A
Moulin et al,[18] 2016	Leukoaraiosis	Present=83%, Absent=17%	Present=60%, Absent=40% (N=54 missing from study's analysis but this is calculated from N=151)	OR=3.2040, CI=1.5485-6.6298	0.0017	Logistic regression (calculated by review authors)	Yes*	N/A	N/A	N/A	N/A
Rasquin et al,[21] 2004	WML	NS	NS	OR=0.9, CI=0.1-8.5	0.9	Chi-square or Fischer's exact test	No*	N/A	N/A	N/A	N/A
Tang et al,[26] 2004	Leukoaraiosis	Present=29%, Absent= 71%	Present=7%, Absent=93%	OR=1.319, CI=1.159 – 1.501	<0.001	Univariate logistic regression	Yes*	OR=1.226, CI=1.031–1.458	0.021	Multivariate logistic regression	Yes*

*finding visualised in the harvest plot (Figure 2)
Abbreviations: N/A, not applicable, NS, not stated

eTable 7.2: PRESENCE of WML and post-stroke COGNITIVE IMPAIRMENT

				Unadjusted analysis				Adjusted analysis			
**Study**	**Neuroimaging feature**	**Developed PSCI**	**Not developed PSCI**	**Effect size**	**Sig. level**	**Statistical test**	**Associated with PSCI**	**Effect size**	**Sig. level**	**Statistical test**	**Associated with PSCI**
Jacquin et al,[10] 2014	Leukoaraiosis	Present=40%, Absent=60%	Present=18%, Absent=82%	OR=3.06, CI=1.64-5.71	0.0005	Logistic regression	Yes*	NS	NS	Multivariable model	No*
Kase et al,[11] 1998	Periventricular lucencies	Present=NS, Absent=NS	Present=NS, Absent=NS	NS	Presence of PVL >0.05, Absence of PVL >0.05	Two sample t-test (MMSE pre-stroke vs post stroke)	No*	N/A	N/A	N/A	N/A
Mehrabian et al,[16] 2015	Periventricular changes	N/A	N/A	Refer to study paper for the beta and t-statistic for each neuropsychological measure	Refer to study paper for the p-value for each neuropsychological measure	Simultaneous multiple regression analyses	No*	N/A	N/A	N/A	N/A
Rasquin et al,[21] 2004	WML	NS	NS	OR=3.9, CI=1.2-12.0	0.02	Chi-square or Fischer's exact test	Yes*	N/A	N/A	N/A	N/A
Saini et al,[23] 2014	Significant WML	N/A	N/A	N/A	N/A	N/A	N/A	OR=3.13 CI=1.27 – 7.70	.01	Logistic regression	Yes*
Schmidt et al,[24] 1993	Leukoaraiosis	Present=20%, Absent=80%	Present=23%, Absent=77%	OR=5, CI=1.26-19.84	0.02	Logistic regression (calculated by review authors)	Yes*	N/A	N/A	N/A	N/A
Thein et al,[27] 2007	Leukoaraiosis	Present=67%, Absent=33%	Present=36%, Absent=64%	OR=3.53, CI=1.19-10.49	0.03	Chi-square	Yes*	N/A	N/A	N/A	N/A

*finding visualised in the harvest plot (Figure 2)
Abbreviations: N/A, not applicable, NS, not stated

eTable 7.3: SEVERITY of WML and post-stroke DEMENTIA

				Unadjusted analysis				Adjusted analysis			
**Study**	**Neuroimaging feature**	**Developed PSD**	**Not developed PSD**	**Effect size**	**Sig. level**	**Statistical test**	**Associated with PSD**	**Effect size**	**Sig. level**	**Statistical test**	**Associated with PSD**
Biffi et al,[5] 2016	White matter disease (0: none or mild; 1: moderate; 2: severe)	NS	NS	NS	<0.05	Log-rank test	Yes*	HR=1.70, CI=1.07-2.71	0.03	Cox proportional hazards regression model	Yes*
Mackowiak-Cordoliani et al,[15] 2003	Leukoaraiosis score	Median=1, Range=0-3	Median=0, Range=0-3	RR=2.09, CI=1.05-4.13	<0.05	Kaplan-Meier survival analysis using the log rank test	Yes*	RR=1.45, CI=1.01-2.06	0.4	Cox proportional hazards regression analysis	No*
Lin et al,[13] 1998	Subcortical WML (0: none, 1: punctate foci, 2: small confluent areas, 3: large confluent areas)	0=4%, 1=24%, 2=48%, 3=22% (1 participant missing from study's analysis)	0=10%, 1=68%, 2=22%, 3=0	NS	<0.001	Exact 2 x k table test for trend	Yes	NS	NS	NS	No
Lin et al,[13] 1998	Overall WML (0: none, 1. punctate lucencies at the tips of the frontal or occipital horns or both, 2: lucencies more extensive but confined to the subependmal region, 3: lucencies more extensive and seen in the subcortical white matter outside the immediate periventricular area, 4: coalescing subcortical white matter lucencies, 5. extensive coalescing subcortical white matter lucencies)	0=4%, 1=6%, 2=20%, 3=26%, 4=38%, 5=6%	0=8%, 1=22%, 2=48%, 3=18%, 4=4%, 5=0%	NS	<0.001	Exact 2 x k table test for trend	Yes*	NS	p<0.0001	Stepwise logistic regression	Yes*

				Unadjusted analysis				Adjusted analysis			
**Study**	**Neuroimaging feature**	**Developed PSD**	**Not developed PSD**	**Effect size**	**Sig. level**	**Statistical test**	**Associated with PSD**	**Effect size**	**Sig. level**	**Statistical test**	**Associated with PSD**
Lin et al,[13] 1998	Periventricular changes (0: none, 1: hyperintensity, capping frontal poles and/or smooth pencil-thin halo around ventricle, 2: thick, smooth halos around ventricles, 3: irregular hyperintensity extending into deep white matter, 4: marked extension of hyperintensity into deep white matter)	0=12%, 1=6%, 2=26%, 3=52%, 4=4%	0=8%, 1=28%, 2=48%, 3=16%, 4=0%	NS	<0.001	Exact 2 x k table test for trend	Yes	NS	NS	NS	No
Loeb et al,[14] 1992	Frequency of leukoaraiosis	NS	NS	NS	Not significant	t-test	No	N/A	N/A	N/A	N/A
Loeb et al,[14] 1992	Degree of leukoaraiosis (grade 3: continuous periventricular leukoaraiosis, grade 4; continuous periventricular leukoaraiosis extending into the corona radiata)	NS	NS	NS	Not significant	t-test	No*	N/A	N/A	N/A	N/A
Moulin et al,[18] 2016	White matter hyperintensities (0: none, 1:punctate, 2: early confluent, 3:confluent)	0=17%, 1=29%, 2=25%, 3=29%	0=39%, 1=30%, 2=22%, 3=6% (Study missing data for 3%)	N/A	N/A	N/A	N/A	SHR=2.88, CI=1.63-5.07 (for score ≥3)	0.0003 (for score ≥3)	Backward stepwise multivariate regression	**Yes** (for score ≥3)*

*finding visualised in the harvest plot (Figure 2)
Abbreviations: N/A, not applicable, NS, not stated

eTable 7.4: SEVERITY of WML and post-stroke COGNITIVE IMPAIRMENT

				Unadjusted analysis				Adjusted analysis			
**Study**	**Neuroimaging feature**	**Developed PSD**	**Not developed PSD**	**Effect size**	**Sig. level**	**Statistical test**	**Associated with PSD**	**Effect size**	**Sig. level**	**Statistical test**	**Associated with PSD**
Appleton et al,[3] 2020	Leukoaraiosis score (of 4), median (IQR)	N/A	N/A	N/A	N/A	N/A	N/A	Refer to study for each cognitive measure	Refer to study for each cognitive measure	Multiple linear regression	Associated with t-MMSE, TICS-M, verbal fluency*
Renjen et al,[22] 2015	White matter change score (0,1,2)	CIND Mean= 1.42, Dementia Mean= 1.26	Mean=1.42	NS	1	Student's t-test, Mann-Whitney U test, Fisher's exact test, and Chi-square test	No*	N/A	N/A	N/A	N/A

*finding visualised in the harvest plot (Figure 2)
Abbreviations: N/A, not applicable, NS, not stated

Supplement 8: Pre-existing stroke lesions

eTable 8.1: PRESENCE of pre-existing stroke lesions and post-stroke DEMENTIA

				**Unadjusted analysis**				**Adjusted analysis**			
**Study**	**Neuroimaging feature**	**Developed PSD**	**Not developed PSD**	**Effect size**	**Sig. level**	**Statistical test**	**Associated with PSD**	**Effect size**	**Sig. level**	**Statistical test**	**Associated with PSD**
Bornstein et al,[6] 1996	Silent brain infarctions	Present=39%, Absent=61%	Present=33%, Absent=67%	OR=1.3, CI=0.6-3.0	0.5	Chi-square	No*	N/A	N/A	N/A	N/A
Mackowiak-Cordoliani et al,[15] 2003	Silent infarcts	Present=41%, Absent=59%	Present=20%, Absent=80%	RR=2.09, CI=1.05-4.13	<0.05	Kaplan-Meier survival analysis using the log rank test	Yes*	NS	>0.05	Cox proportional hazards regression analysis	No*
Rasquin et al,[21] 2004	Silent infarcts	Present=NS, Absent=NS	Present=NS, Absent=NS	OR=5.6, CI=1.4-22.5	0.01	Chi-square or Fischer's exact test	Yes*	N/A	N/A	N/A	N/A

*finding visualised in the harvest plot (Figure 2)
Abbreviations: N/A, not applicable, NS, not stated

eTable 8.2: PRESENCE of pre-existing stroke lesions and post-stroke COGNITIVE IMPAIRMENT

				Unadjusted analysis				Adjusted analysis			
**Study**	**Neuroimaging feature**	**Developed PSD/PSCI**	**Not developed PSD/PSCI**	**Effect size**	**Sig. level**	**Statistical test**	**Associated with PSCI**	**Effect size**	**Sig. level**	**Statistical test**	**Associated with PSCI**
Jacquin et al,[10] 2014	Silent infarcts	Present=30%, Absent=70%	Present=9%, Absent=91%	OR=4.05 CI=1.92-8.58	0.0003	Logistic regression	**Yes***	OR=3.31, CI=1.32-8.29	0.011	Multivariable model	**Yes***
Kase et al,[11] 1998	Silent infarcts	NS	NS	NS	Present >0.05, Absent >0.05	Two sample t-test (MMSE pre-stroke vs post stroke)	No*	N/A	N/A	N/A	N/A
Rasquin et al,[21] 2004	Silent infarcts	Present=NS, Absent=NS	Present=NS, Absent=NS	OR=1.4, CI=0.6-3.1	0.47	Chi-square or Fischer's exact test	No*	N/A	N/A	N/A	N/A
Renjen et al,[22] 2015	Silent infarcts	'Cognitive impairment was more frequent in patients with… silent infarcts (88.8%)'	NS	NS	0.694	Student's t-test, Mann-Whitney U test, Fisher's exact test, and Chi-square test	No*	N/A	N/A	N/A	N/A
Saini et al,[23] 2014	Chronic infarcts	NS	NS	N/A	N/A	N/A	N/A	OR=2.44, CI=1.27-4.70	0.1	Logistic regression	No*

*finding visualised in the harvest plot (Figure 2)
Abbreviations: N/A, not applicable, NS, not stated

eTable 8.3: NUMBER of pre-existing stroke lesions and post-stroke DEMENTIA

				Unadjusted analysis				Adjusted analysis			
Study	Neuroimaging feature	Developed PSD	Not developed PSD	Effect size	Sig. level	Statistical test	Associated with PSD	Effect size	Sig. level	Statistical test	Associated with PSD
Bornstein et al,[6] 1996	Number of silent brain infarctions	Single=64%, Multiple=36%	Single=61%, Multiple=39%	NS	NS	NS	No*	N/A	N/A	N/A	N/A
Klimkowicz-Mrowiec et al,[12] 2006	Mean number of old infarcts	Mean=1.2, SD=0.9	Mean=0.9, SD=1.1	NS	0.1	Chi-square	No*	N/A	N/A	N/A	N/A

*finding visualised in the harvest plot (Figure 2)
Abbreviations: N/A, not applicable, NS, not stated

eTable 8.4: LOCATION of pre-existing stroke lesions and post-stroke DEMENTIA

				Unadjusted analysis				Adjusted analysis			
Study	Neuroimaging feature	Developed PSD	Not developed PSD	Effect size	Sig. level	Statistical test	Associated with PSD	Effect size	Sig. level	Statistical test	Associated with PSD
Bornstein et al,[6] 1996	Location of silent brain infarctions	Lacunar=NS, Cortical=NS	Lacunar=NS Cortical=NS	NS	0.4	Chi-square	No*	N/A	N/A	N/A	N/A

*finding visualised in the harvest plot (Figure 2)
Abbreviations: N/A, not applicable, NS, not stated

eTable 8.5: LOCATION of pre-existing stroke lesions and post-stroke COGNITIVE IMPAIRMENT

				Unadjusted analysis				Adjusted analysis			
**Study**	**Neuroimaging feature**	**Developed PSD**	**Not developed PSD**	**Effect size**	**Sig. level**	**Statistical test**	**Associated with PSCI**	**Effect size**	**Sig. level**	**Statistical test**	**Associated with PSCI**
Gomez-Vierra et al,[8] 2002	Location of old infarcts	Unilateral=15%, Bilateral=25%	Unilateral=4%, Bilateral=20%	RR=1.82, CI=1.26-2.63	0.06	Chi square test	No*	N/A	N/A	N/A	N/A
Appleton et al,[3] 2020	Old infarcts: striatocapsular	N/A	N/A	N/A	N/A	N/A	N/A	Refer to study for each cognitive measure	Refer to study for each cognitive measure	Multiple linear regression	No*
Appleton et al,[3] 2020	Old infarcts: border zone	N/A	N/A	N/A	N/A	N/A	N/A	Refer to study for each cognitive measure	Refer to study for each cognitive measure	Multiple linear regression	No
Appleton et al,[3] 2020	Old infarcts: lacunar	N/A	N/A	N/A	N/A	N/A	N/A	Refer to study for each cognitive measure	Refer to study for each cognitive measure	Multiple linear regression	Associated with verbal fluency
Appleton et al,[3] 2020	Old infarcts: at least 1 of striatocapsule, border zone, lacunar	N/A	N/A	N/A	N/A	N/A	N/A	Refer to study for each cognitive measure	Refer to study for each cognitive measure	Multiple linear regression	Associated with verbal fluency
Saini et al,[23] 2014	Bilateral chronic infarcts	NS	NS	N/A	N/A	N/A	N/A	NS	0.01	Logistic regression	Yes*
Saini et al,[23] 2014	Thalamic chronic infarcts	NS	NS	N/A	N/A	N/A	N/A	NS	0.05	Logistic regression	No
Saini et al,[23] 2014	Subcortical chronic infarcts	NS	NS	N/A	N/A	N/A	N/A	NS	0.02	Logistic regression	Yes

*finding visualised in the harvest plot (Figure 2)
Abbreviations: N/A, not applicable, NS, not stated

Supplement 9: Pathological stroke type

eTable 9.1: Type of stroke and post-stroke DEMENTIA

				Unadjusted analysis				Adjusted analysis			
**Study**	**Neuroimaging feature**	**Developed PSD**	**Not developed PSD**	**Effect size**	**Sig. level**	**Statistical test**	**Associated with PSD**	**Effect size**	**Sig. level**	**Statistical test**	**Associated with PSD**
Barba et al,[4] 2000	Type of stroke	Ischaemic=89%, Haemorrhagic=11%	Ischaemic=88%, Haemorrhagic=12%	OR=0.9, CI=0.3-2	>0.1	Chi-square test	No*	N/A	N/A	N/A	N/A
Mackowiak-Cordoliani et al,[15] 2003	Type of stroke	Ischaemic=94%, Haemorrhagic=6%	Ischaemic=91%, Haemorrhagic=9%	RR=1.63, CI=0.39-6.81	>0.05	Kaplan-Meier survival analysis using the log rank test	No*	N/A	N/A	N/A	N/A
Klimkowicz-Mrowiec et al,[12] 2006	Type of stroke	Ischaemic=86%, Haemorrhagic=14%	Ischaemic=89%, Haemorrhagic=11%	OR=0.8035, CI=0.2962-2.1797	0.6674	Logistic regression (calculated by review authors)	No*	N/A	N/A	N/A	N/A
Tang et al,[26] 2004	Type of stroke	Ischaemic=93%, Haemorrhagic=7%	Ischaemic=92%, Haemorrhagic=8%	OR=0.867, CI=0.281-2.674	0.804	Univariate logistic regression	No*	N/A	N/A	N/A	N/A

*finding visualised in the harvest plot (Figure 2)
Abbreviations: N/A, not applicable, NS, not stated

eTable 9.2: Type of stroke and post-stroke COGNITIVE IMPAIRMENT

				Unadjusted analysis				Adjusted analysis			
**Study**	**Measurement method**	**Developed PSD/PSCI**	**Not developed PSD/PSCI**	**Effect size**	**Sig. level**	**Statistical test**	**Associated with PSCI**	**Effect size**	**Sig. level**	**Statistical test**	**Associated with PSCI**
Jacquin et al,[10] 2014	Type of stroke	Ischaemic=95%, Haemorrhagic=5%	Ischaemic=91%, Haemorrhagic=9%	OR=2.07, CI=0.70-6.18	0.191	Logistic regression	No*	N/A	N/A	N/A	N/A
Renjen et al,[22] 2015	Type of stroke	Ischaemic= 69%, Haemorrhagic= 31%	Ischaemic= 86%, Haemorrhagic= 14%	OR=0.38, CI=0.07-1.99	0.25	Logistic regression (calculated by review authors)	No*	N/A	N/A	N/A	N/A
Zhang et al,[28] 2012	Type of stroke	Present= 89%, Absent= 11%	Present= 93%, Absent= 7%	OR=0.63, CI=0.34-1.15	0.13	Logistic regression (calculated by review authors)	No*	N/A	N/A	N/A	N/A

*finding visualised in the harvest plot (Figure 2)
Abbreviations: N/A, not applicable, NS, not stated

Supplement 10: Acute stroke features

eTable 10.1: LOCATION of stroke and post-stroke DEMENTIA

				Unadjusted analysis				Adjusted analysis			
**Study**	**Neuroimaging feature**	**Developed PSD**	**Not developed PSD**	**Effect size**	**Sig. level**	**Statistical test**	**Associated with PSD**	**Effect size**	**Sig. level**	**Statistical test**	**Associated with PSD**
Barba et al,[4] 2000	Mechanism of ischemic stroke	Thrombotic=79%, Embolic=21%	Thrombotic=89%, Embolic=11%	OR=2.2, CI=1.1-4.9	P<0.1	Chi-square test	Yes	N/A	N/A	N/A	N/A
Barba et al,[4] 2000	Location of stroke	Left carotid=52%, Right carotid=36%, Vertibrobasilar=11%, Undefinate=1%	Left carotid=45%, Right carotid=34%, Vertibrobasilar=20%, Undefinate=1%	OR=0.9, CI=0.5-1.6 (left vs right)	P>0.1 (left vs right)	Chi-square test	No	N/A	N/A	N/A	N/A
Biffi et al,[5] 2016	ICH location: lobar	NS	NS	N/A	N/A	N/A	N/A	HR=1.33, CI=0.25-2.71	.74	Cox proportional hazards regression model	No
Klimkowicz-Mrowiec et al,[12] 2006	Side of stroke lesion	Left=52%, Right=48%	Left=43%, Right=57%	NS	0.56	Chi-square	No	N/A	N/A	N/A	N/A
Lin et al,[13] 1998	Cortical involvement (cortical, border zone, mixed) or subcortical involvement (lacune, subcortical)	Cortical=72%, Subcortical=28%	Cortical=34%, Subcortical=66%	NS	p<0.001	Exact 2 x 2 table test	Yes (cortical)	NS	>0.05	Stepwise logistic regression	No
Lin et al,[13] 1998	Cortical involvement: cortical infarct (that measures >15mm^3)	Present=12%, Absent=88%	Present=14%, Absent=86%	NS	>0.05	Exact 2 x 2 table test	No	NS	>0.05	Stepwise logistic regression	No
Lin et al,[13] 1998	Cortical involvement: border zone (cortical or subcortical infarct that is located within the boundary zone of major cerebral arteries)	Present=6%, Absent=94%	Present=2%, Absent=98%	NS	>0.05	Exact 2 x 2 table test	No	NS	>0.05	Stepwise logistic regression	No
Lin et al,[13] 1998	Cortical involvement: Mixed (cortical or subcortical infarct	Present=54%, Absent=46%	Present=18%, Absent=82%	NS	<0.001	Exact 2 x 2 table test	Yes	NS	>0.05	Stepwise logistic regression	No

				Unadjusted analysis				Adjusted analysis			
**Study**	**Neuroimaging feature**	**Developed PSD**	**Not developed PSD**	**Effect size**	**Sig. level**	**Statistical test**	**Associated with PSD**	**Effect size**	**Sig. level**	**Statistical test**	**Associated with PSD**
	mixed with lacunae)										
Lin et al,[13] 1998	Subcortical involvement: Lacune (infarct that measures >15mm^3 in the vascular territory of a deep penetrating artery)	Present=24%, Absent=76%	Present=58%, Absent=42%	NS	<0.001	Exact 2 x 2 table test	Yes	NS	>0.05	Stepwise logistic regression	No
Lin et al,[13] 1998	Subcortical involvement: Subcortical (subcortical infarct that measures >15mm^3 and is not a border zone infarct)	Present=4%, Absent=96%	Present=8%, Absent=92%	NS	>0.05	Exact 2 x 2 table test	No	NS	>0.05	Stepwise logistic regression	No
Lin et al,[13] 1998	Laterality of lacunar infarction: Basal ganglion	None=38%, Right=2%, Left=4%, Bilateral=56%	None=56%, Right=14%, Left=10%, Bilateral=20%	NS	<0.001	Exact 2 x 4 table test for trend	Yes	NS	>0.05	Stepwise logistic regression	No
Lin et al,[13] 1998	Laterality of lacunar infarction: Thalamus	None=50%, Right=6%, Left=12%, Bilateral=32%	None=82%, Right=2%, Left=0%, Bilateral=0%	NS	<0.01	Exact 2 x 4 table test for trend	Yes	NS	>0.05	Stepwise logistic regression	No
Lin et al,[13] 1998	Laterality of lacunar infarction: Brain stem	None=96%, Right=4%, Left=0%, Bilateral=0%	None=86%, Right=6%, Left=8%, Bilateral=0%	NS	>0.05	Exact 2 x 4 table test for trend	No	NS	>0.05	Stepwise logistic regression	No
Lin et al,[13] 1998	Laterality of lacunar infarction: Cerebellum	None=98%, Right=2%, Left=0%, Bilateral=0%	None=100%, Right=0%, Left=0%, Bilateral=0%	NS	>0.05	Exact 2 x 4 table test for trend	No	NS	>0.05	Stepwise logistic regression	No
Lin et al,[13] 1998	Laterality of lacunar infarction: Internal capsule & corona radiata	None=62%, Right=12%, Left=4%, Bilateral=22%	None=60%, Right=24%, Left=10%, Bilateral=3%	NS	>0.05	Exact 2 x 4 table test for trend	No	NS	>0.05	Stepwise logistic regression	No
Rasquin et al,[21] 2004	Side of stroke	Left=NS, Right=NS	Left=NS, Right=NS	OR=2.1, CI=0.6-7.2	0.23	chi-square / Fischer's Exact test	No	N/A	N/A	N/A	N/A

				Unadjusted analysis				Adjusted analysis			
**Study**	**Neuroimaging feature**	**Developed PSD**	**Not developed PSD**	**Effect size**	**Sig. level**	**Statistical test**	**Associated with PSD**	**Effect size**	**Sig. level**	**Statistical test**	**Associated with PSD**
Rasquin et al,[21] 2004	Stroke type	Territorial=NS, Lacunar=NS	Territorial=NS, Lacunar=NS	OR=4.5, CI=1.2-16.8	0.02	chi-square / Fischer's Exact test	Yes	OR=6.2, CI=0.9-38.7	>0.05	Multivariate logistic regression	No
Tang et al,[26] 2004	Subtype of infarct: cortical	Present=3%, Absent=97%	Present=19%, Absent=81%	OR=2.134, CI=1.103-4.129	0.024	Univariate logistic regression	Yes	NS	NS	NS	NS
Tang et al,[26] 2004	Subtype of infarct: subcortical	Present=18%, Absent=82%	Present=22%, Absent=88%	OR=0.904, CI=0.619-1.321	0.602	Univariate logistic regression	No	N/A	N/A	N/A	N/A
Tang et al,[26] 2004	Subtype of infarct: brainstem	Present=2%, Absent=98%	Present=7%, Absent=93%	OR=0.648, CI=0.327-1.284	0.214	Univariate logistic regression	No	N/A	N/A	N/A	N/A
Tang et al,[26] 2004	Subtype of infarct: lacunar	Present=60%, Absent=40%	Present=59%, Absent=41%	OR=1.020, CI=0.875-1.188	0.804	Univariate logistic regression	No	N/A	N/A	N/A	N/A
Tang et al,[26] 2004	Subtype of infarct: cerebellar	Present=2%, Absent=98%	Present=3%, Absent=97%	OR=0.892, CI=0.584-1.362	0.596	Univariate logistic regression	No	N/A	N/A	N/A	N/A
Tang et al,[26] 2004	Bilateral lesions	Present=55%, Absent=45%	Present=30%, Absent=70%	OR=2.769, CI=1.512-5.073	0.001	Univariate logistic regression	Yes	OR=2.935, CI=1.154-7.466	0.024	Multivariate logistic model	Yes
Tang et al,[26] 2004	Vascular territory involvement: anterior cerebral artery	Present=9%, Absent=91%	Present=8%, Absent=92%	OR=1.213, CI=0.426-3.452	0.717	Univariate logistic regression	No	N/A	N/A	N/A	N/A
Tang et al,[26] 2004	Vascular territory involvement: middle cerebral artery	Present=91%, Absent=9%	Present=67%, Absent=33%	OR=4.732, CI=1.805-12.405	0.002	Univariate logistic regression	Yes	OR=4.099, CI=1.162-14.461	0.028	Forward stepwise multivariate logistic regression	Yes
Tang et al,[26] 2004	Vascular territory involvement: posterior cerebral artery	Present=17%, Absent=83%	Present=30%, Absent=70%	OR=0.472, CI=0.218-1.023	0.057	Univariate logistic regression	No	N/A	N/A	N/A	N/A
Tang et al,[26] 2004	Vascular territory involvement: internal carotid artery	Present=6%, Absent=94%	Present=7%, Absent=93%	OR=0.800, CI=0.223-2.871	0.732	Univariate logistic regression	No	N/A	N/A	N/A	N/A
Moulin et al,[18] 2016	Location of haemorrhagic stroke	Lobar=49%, Non-lobar=51%	Lobar=30%, Non-lobar=70%	OR=2.2955, CI=1.2570-4.1922	0.0068	Logistic regression (calculated by review authors)	Yes	NS	NS	Backward stepwise multivariable analysis	No

*finding visualised in the harvest plot (Figure 2). Abbreviations: N/A, not applicable, NS, not stated

eTable 10.2: LOCATION of stroke and post-stroke COGNITIVE IMPAIRMENT

				Unadjusted analysis				Adjusted analysis			
**Study**	**Neuroimaging feature**	**Developed PSCI**	**Not developed PSCI**	**Effect size**	**Sig. level**	**Statistical test**	**Associated with PSCI**	**Effect size**	**Sig. level**	**Statistical test**	**Associated with PSCI**
Appleton et al,[3] 2020	Visible infarction	N/A	N/A	N/A	N/A	N/A	N/A	Refer to study for each cognitive measure	Refer to study for each cognitive measure	Multiple linear regression	Associated with t-MMSE, TICS-M, verbal fluency
Appleton et al,[3] 2020	Visible haemorrhage	N/A	N/A	N/A	N/A	N/A	N/A	Refer to study for each cognitive measure	Refer to study for each cognitive measure	Multiple linear regression	Associated with verbal fluency
Appleton et al,[3] 2020	Lacunar	N/A	N/A	N/A	N/A	N/A	N/A	Refer to study for each cognitive measure	Refer to study for each cognitive measure	Multiple linear regression	Associated with verbal fluency
Appleton et al,[3] 2020	Parenchymal haemorrhage	N/A	N/A	N/A	N/A	N/A	N/A	Refer to study for each cognitive measure	Refer to study for each cognitive measure	Multiple linear regression	Associated with verbal fluency
Appleton et al,[3] 2020	Lobar or cerebellar	N/A	N/A	N/A	N/A	N/A	N/A	Refer to study for each cognitive measure	Refer to study for each cognitive measure	Multiple linear regression	No
Appleton et al,[3] 2020	Deep	N/A	N/A	N/A	N/A	N/A	N/A	Refer to study for each cognitive measure	Refer to study for each cognitive measure	Multiple linear regression	Associated with verbal fluency
Alexandrova et al,[1] 2016	Location of stroke	Hemispherical= 90%, Lacunar=10%, Stem=0%	Hemispherical= 78%, Lacunar= 4%, Stem= 19%	NS	0.126	General linear model repeated measures analysis / Friedman test	No	N/A	N/A	N/A	N/A
Rasquin et al,[21] 2004	Stroke type	Territorial=NS, Lacunar=NS	Territorial=NS, Lacunar=NS	OR=1.3, CI=0.6-2.8	0.45	Chi-square / Fischer's Exact test	No	N/A	N/A	N/A	N/A
Mehrabian et al,[16] 2015	Presence of Basal ganglia lesions	N/A	N/A	Refer to study paper for the beta and t-statistic for	Refer to study paper for the p-value for each	Simultaneous multiple regression	No	N/A	N/A	N/A	N/A

				Unadjusted analysis				Adjusted analysis			
Study	Neuroimaging feature	Developed PSCI	Not developed PSCI	Effect size	Sig. level	Statistical test	Associated with PSCI	Effect size	Sig. level	Statistical test	Associated with PSCI
				each neuropsychological measure	neuropsychological measure						
Schmidt et al,[24] 1993	Location of infarct: Frontal lobe (%)	Present=33%, Absent=67%	Present=12%, Absent=88%	NS	NS	Fischer's Exact test	No	N/A	N/A	N/A	N/A
Schmidt et al,[24] 1993	Location of infarct: Parietal lobe (%)	Present=60%, Absent=40%	Present=46%, Absent=54%	NS	NS	Fischer's Exact test	No	N/A	N/A	N/A	N/A
Schmidt et al,[24] 1993	Location of infarct: Temporal lobe (%)	Present=67%, Absent=33%	Present=12%, Absent=88%	NS	P<0.001 vs controls, P<0.05 vs transient cognitive impairment	Fischer's Exact test	Yes	Beta=1.4; SE (beta)=0.47	0.47	<0.01	Yes
Schmidt et al,[24] 1993	Location of infarct: Occipital lobe (%)	Present=0%, Absent=100%	Present=8%, Absent=92%	NS	NS	Fischer's Exact test	No	N/A	N/A	N/A	N/A
Schmidt et al,[24] 1993	Location of infarct: Basal ganglia (%)	Present=40%, Absent=60%	Present=50%, Absent=50%	NS	NS	Fischer's Exact test	No	N/A	N/A	N/A	N/A
Schmidt et al,[24] 1993	Location of infarct: Thalamus (%)	Present=20%, Absent=80%	Present=0%, Absent=100%	NS	NS	Fischer's Exact test	No	N/A	N/A	N/A	N/A
Schmidt et al,[24] 1993	Location of infarct: Cerebellum (%)	Present=7%, Absent=93%	Present=8%, Absent=92%	NS	NS	Fischer's Exact test	No	N/A	N/A	N/A	N/A
Gomez-Vierra et al,[8] 2002	Acute infarction	Unilateral=73%, Bilateral=27%	Unilateral=92%, Bilateral=8%	RR=1.82; CI=1.26-2.63	0.00	Chi square test	Yes	N/A	N/A	N/A	N/A
Gomez-Vierra et al,[8] 2002	Location of acute infarction	Right frontal lobe=5%, Left frontal lobe=10%, Right parietal lobe=1%, Left parietal lobe=18%, Right temporal lobe=9%, Left temporal lobe=14%, Right occipital lobe=2%, Left occipital lobe=10%,	Right frontal lobe=11%, Left frontal lobe=5%, Right parietal lobe=5%, Left parietal lobe=6%, Right temporal lobe=14%, Left temporal lobe=1%, Right occipital lobe=9%, Left occipital lobe=6%,	NS	0.07	Chi square	No	N/A	N/A	N/A	N/A

				Unadjusted analysis				Adjusted analysis			
**Study**	**Neuroimaging feature**	**Developed PSCI**	**Not developed PSCI**	**Effect size**	**Sig. level**	**Statistical test**	**Associated with PSCI**	**Effect size**	**Sig. level**	**Statistical test**	**Associated with PSCI**
		Oval center=0%, Internal capsule=5%, Thalamus=0%, Caudate nucleus=0%, Brainstem=8%, Cerebellum=7%, Periventricular region=10%	Oval centre=1%, Internal capsule=7%, Thalamus=6%, Caudate nucleus=2%, Brainstem=7%, Cerebellum=11% , Periventricular region=8%								
Gomez-Vierra et al,[8] 2002	Vascular territory	Left internal carotid artery=3%, Right internal carotid artery=2%, Left anterior cerebral artery=7%, Right anterior cerebral artery=9%, Left middle cerebral artery=51%, Right middle cerebral artery=16%, Left posterior cerebral artery=5%, Right posterior cerebral artery=2%, Vertebrobasilar artery=5%	Left internal carotid artery=2%, Right internal carotid artery=0%, Left anterior cerebral artery=7%, Right anterior cerebral artery=9%, Left middle cerebral artery=43%, Right middle cerebral artery=22%, Left posterior cerebral artery=6%, Right posterior cerebral artery 7%, Vertebrobasilar artery=4%	NS	0.52	Chi square test	No	N/A	N/A	N/A	N/A
Jacquin et al,[10] 2014	Location of stroke	NS	NS	NS	NS	Logistic regression	No	N/A	N/A	N/A	N/A
House et al,[9] 1990	Location of stroke	Left=NS, Right=NS	Left=NS, Right=NS	z=1.62	0.11	Mann Whitney	No	N/A	N/A	N/A	N/A
Kase et al,[11] 1998	Stroke laterality	Left=NS, Right=NS	Left=NS, Right=NS	NS	Left side=>0.05, Right side=>0.05	Two sample t-test (MMSE pre-stroke vs post stroke)	No	N/A	N/A	N/A	N/A

				Unadjusted analysis				Adjusted analysis			
**Study**	**Neuroimaging feature**	**Developed PSCI**	**Not developed PSCI**	**Effect size**	**Sig. level**	**Statistical test**	**Associated with PSCI**	**Effect size**	**Sig. level**	**Statistical test**	**Associated with PSCI**
Kase et al,[11] 1998	Location superficial (affected the cerebral cortex and the subcortical white matter) or deep (involving the basal ganglia, internal capsule or thalamus)	NS	NS	NS	Superficial=>0.05, Deep >0.05	Two sample t-test (MMSE pre-stroke vs post stroke)	No	N/A	N/A	N/A	N/A
Schmidt et al,[24] 1993	Side of stroke	Left=40%, Right=60%;	Left=38%, Right=62%;	NS	NS	Fisher's Exact test	No	NS	NS	Stepwise backward regression	No
Rasquin et al,[21] 2004	Side of stroke	Left=NS, Right=NS	Left=NS, Right=NS	OR=1.3, CI=0.6-2.6	0.56	Chi-square / Fischer's Exact test	No	N/A	N/A	N/A	N/A
Pinzon et al,[19] 2018	Location of brain lesion: frontal cortex	18%	20%	OR=1.27, CI=0.479-3.143	0.669	Chi-square test	No	N/A	N/A	N/A	N/A
Pinzon et al,[19] 2018	Location of brain lesion: occipital cortex	6%	5%	OR= 1.435; CI=0.275-7.495	0.667	Chi-square test	No	N/A	N/A	N/A	N/A
Pinzon et al,[19] 2018	Location of brain lesion: no cortical lesion	14%	51%	OR=1.67, CI=0.069-0.403	<0.001	Chi-square test	Yes	NS	0.041	Logistic regression	Yes
Pinzon et al,[19] 2018	Location of brain lesion: parietal cortex	40%	22%	OR=4.1, CI=1.69-9.95	0.001	Chi-square test	No	OR=4.87, CI=0.753-31.477	0.096	Logistic regression	No
Pinzon et al,[19] 2018	Location of brain lesion: temporal cortex	22%	2%	OR=16, CI=2.066-123.9	0.001	Chi-square test	Yes	OR=26.102, CI=2.146-317.43	0.01	Logistic regression	Yes
Prodjohardjono et al,[20] 2020	Infarct side	Left=43%, Right=34%, Both=23%	Left=33%, Right=67%, Both=0%	NS	0.018	Chi-square	Yes	Left vs right OR=12.13 (1.39-42.91), Both vs right OR=infinite (0.00-infinite)	Left vs right=0.959, Both vs right=0.955	Multivariate logistic regression	No

				Unadjusted analysis				Adjusted analysis			
**Study**	**Neuroimaging feature**	**Developed PSCI**	**Not developed PSCI**	**Effect size**	**Sig. level**	**Statistical test**	**Associated with PSCI**	**Effect size**	**Sig. level**	**Statistical test**	**Associated with PSCI**
Prodjohardjono et al,[20] 2020	Infarct location	Cortical=9%, Subcortical=83% , Cortical & subcortical=9%	Cortical=5%, Subcortical=95%, Cortical & subcortical=0%	NS	0.317	Chi-square	No	Subcortical vs cortical OR=0.26 (0.01 - infinite), Both vs cortical OR=0.04 (0.00 - infinite)	Subcortical vs cortical=0.991 , Both vs cortical=0.979	Multivariate logistic regression	No
Zhang et al,[28] 2012	Left-hemisphere stroke	Present= 51%, Absent= 49%	Present= 55%, Absent= 45%	OR=0.87, CI=0.61-1.23	0.425	Chi-square test	No	N/A	N/A	N/A	N/A
Zhang et al,[28] 2012	Right-hemisphere stroke	Present= 59%, Absent= 41%	Present= 52%, Absent= 48%	OR=1.33, CI=0.93-1.91	0.116	Chi-square test	No	N/A	N/A	N/A	N/A
Zhang et al,[28] 2012	Lesion location: subcortical	Present= 57%, Absent= 43%	Present= 57%, Absent= 43%	OR=0.99, 0.69-1.1	0.955	Chi-square test	No	N/A	N/A	N/A	N/A
Zhang et al,[28] 2012	Lesion location: cortex	Present= 35%, Absent= 65%	Present= 27%, Absent= 73%	OR=1.48, CI=1.01-2.16	0.044	Chi-square test	Yes	OR=1.55, CI=1.04-2.31	0.033	Multivariable stepwise logistic regression	Yes
Zhang et al,[28] 2012	Lesion location: infratentorial	Present= 19%, Absent=81%	Present=25%, Absent= 75%	OR=0.71, CI=0.45-1.1	0.122	Chi-square test	No	N/A	N/A	N/A	N/A
Renjen et al,[22] 2015	Side of lesion (left vs right)	'Cognitive impairment was more frequent in patients with left-sided lesions (80.7%)	NS	NS	0.176	Student's t-test, Mann-Whitney U test, Fisher's exact test, and Chi-square test	No	N/A	N/A	N/A	N/A
Renjen et al,[22] 2015	Location of lesion	Subcortical= 25, Cortical= 11	Subcortical= 7, cortical= 7	NS	NS	Student's t-test, Mann-Whitney U test, Fisher's exact test, and Chi-square test	No	N/A	N/A	N/A	N/A
Sundar et al,[25] 2010	Pure cortical vs pure subcortical (includes both single and multiple	Pure cortical=38%; Pure subcortical=40%; Mixed=21%	Pure cortical=19%; Pure subcortical=35%; Mixed=46%	NS	NS	NS	Yes	N/A	N/A	N/A	N/A

				Unadjusted analysis				Adjusted analysis			
**Study**	**Neuroimaging feature**	**Developed PSCI**	**Not developed PSCI**	**Effect size**	**Sig. level**	**Statistical test**	**Associated with PSCI**	**Effect size**	**Sig. level**	**Statistical test**	**Associated with PSCI**
	infarcts within each category)										

*finding visualised in the harvest plot (Figure 2)
Abbreviations: N/A, not applicable, NS, not stated

eTable 10.3: SIZE of lesion and post-stroke DEMENTIA

				Unadjusted analysis				Adjusted analysis			
**Study**	**Neuroimaging feature**	**Developed PSD/PSCI**	**Not developed PSD/PSCI**	**Effect size**	**Sig. level**	**Statistical test**	**Associated with PSD**	**Effect size**	**Sig. level**	**Statistical test**	**Associated with PSD**
Loeb et al,[14] 1992	Lesion volume	Mean=6.80ml, SD=0.68ml	Mean=6.47ml, SD=0.63ml	NS	Not significant	t-test	No	N/A	N/A	N/A	N/A
Moulin et al,[18] 2016	Haemorrhage volume	Median=9ml, IQR=3-19	Median=6ml, IQR=2.22	N/A	N/A	N/A	N/A	NS	NS	Backward stepwise multivariable analysis	No

*finding visualised in the harvest plot (Figure 2)
Abbreviations: N/A, not applicable, NS, not stated

eTable 10.4: SIZE of lesion and post-stroke COGNITIVE IMPAIRMENT

				Unadjusted				Adjusted			
**Study**	**Neuroimaging feature**	**Developed PSCI**	**Not developed PSCI**	**Effect size**	**Sig. level**	**Statistical test**	**Associated with PSCI**	**Effect size**	**Sig. level**	**Statistical test**	**Associated with PSCI**
Gomez-Vierra et al,[8] 2002	Larger stroke size	Up to 50mL=41%, Over 50mL=59%	Up to 50mL=62%, Over 50mL=38%	RR=2.44, CI=1.51-3.92	0.00	chi square test	Yes	RR=1.373, CI=0.699-2.697	0.357	Multiple logistic regression	No
House et al,[9] 1990	Lesion volume	NS	NS	Rs=-0.32, N=64	0.005	unlear	Yes	N/A	N/A	N/A	N/A
Schmidt et al,[24] 1993	Infarct volume in the first week Mean (SD)	Persistent cognitive impairment=25.5 (37.1)	Transient cognitive impairment=12.2 (16.3), No cognitive impairment=3.9 (6.8)	NS	NS	Kruskal Wallis test	No	NS	NS	Stepwise backward regression	No
Kase et al,[11] 1998	Stroke size: Large (involved more than one half of a cerebral lobe) or Small (lacunar infarct or involved less than one half of a cerebral lobe)	Small=NS, Large=NS	Small=NS, Large=NS	NS	Large <0.05, Small >0.05	Two sample t-test (MMSE pre-stroke vs post stroke)	Yes	N/A	N/A	N/A	N/A
Kase et al,[11] 1998	Stroke size: left side only	Small=NS, Large=NS	Small=NS, Large=NS	NS	Small >0.05, Large <0.05	Two sample t-test (MMSE pre-stroke vs post stroke)	Yes	N/A	N/A	N/A	N/A
Kase et al,[11] 1998	Stroke size: right side only	Small=NS, Large=NS	Small=NS, Large=NS	NS	Small >0.05, Large>0,05	Two sample t-test (MMSE pre-stroke vs post stroke)	No	N/A	N/A	N/A	N/A
Andersen et al,[2] 1996	Lesion size	NS	NS	N/A	N/A	N/A	N/A	Percent of observed variance=1	0.07	Stepwise multiple regression analysis	No
Prodjohardjono et al,[20] 2020	Infarct volume	≥0.054=86%, <0.054=14%	≥0.054=38%, <0.054=62%	OR=9.75, CI=2.67-35.53	<0.001	Bivariate analysis	Yes	OR=7.71, CI=1.39-42.91	0.019	Multivariate logistic regression	Yes

				Unadjusted				Adjusted			
Study	Neuroimaging feature	Developed PSCI	Not developed PSCI	Effect size	Sig. level	Statistical test	Associated with PSCI	Effect size	Sig. level	Statistical test	Associated with PSCI
Prodjohardjono et al,[20] 2020	Infarct volume	Median=0.19ml, Range=0.02-16.08	Median=0.04ml, Range=0.003-1.49	NS	0.003	Independent t-test/Mann Whitney U	Yes	N/A	N/A	N/A	N/A
Renjen et al,[22] 2015	Size of lesion	NS	NS	NS	NS	Student's t-test, Mann-Whitney U test, Fisher's exact test, and Chi-square test	No	N/A	N/A	N/A	N/A

*finding visualised in the harvest plot (Figure 2)
Abbreviations: N/A, not applicable, NS, not stated

eTable 10.5: NUMBER of lesions and post-stroke DEMENTIA

				Unadjusted analysis				Adjusted analysis			
Study	Neuroimaging feature	Developed PSD/PSCI	Not developed PSD/PSCI	Effect size	Sig. level	Statistical test	Associated with PSD	Effect size	Sig. level	Statistical test	Associated with PSD
Barba et al,[4] 2000	Multiple lesions	Single=44%, Multiple=44% (CT not available/no definite vascular lesion=12%)	Single=62%, Multiple=28% (CT not available/no definite vascular lesion=10%)	OR=2.2; CI=1.2-4	P<0.1	Chi-square test	Yes	N/A	N/A	N/A	N/A
Tang et al,[26] 2004	Number of lesions	Mean=1.7, SD=1.0	Mean=1.5, SD=0.9	OR=1.609, CI=1.160-2.232	0.004	Univariate logistic regression	Yes	N/A	N/A	N/A	N/A
Lin et al,[13] 1998	Number of lacunae: Basal ganglion	0=38%, 1-4=20%, 5-9=40%, ≥10=2%	0=56%, 1-4=36%, 5-9=8%, ≥10=0%	NS	<0.001	Exact 2 x 4 table test	Yes	NS	>0.05	Stepwise logistic regression	No
Lin et al,[13] 1998	Number of lacunae: Thalamus	0=50% 1-4=44%, 5-9=6%, ≥10=0%	0=82%, 1-4=18%, 5-9=0%, ≥10=0%	NS	<0.01	Exact 2 x 4 table test for trend	Yes	NS	<0.05	Stepwise logistic regression	Yes
Lin et al,[13] 1998	Number of lacunae: Brain stem	0=96%; 1-4=4%; 5-9=0%; ≥10=0%.	0=88%; 1-4=12%; 5-9=0%; ≥10=0%.	NS	>0.05	Exact 2 x 4 table test for trend	No	NS	>0.05	Stepwise logistic regression	No

				Unadjusted analysis				Adjusted analysis			
**Study**	**Neuroimaging feature**	**Developed PSD/PSCI**	**Not developed PSD/PSCI**	**Effect size**	**Sig. level**	**Statistical test**	**Associated with PSD**	**Effect size**	**Sig. level**	**Statistical test**	**Associated with PSD**
Lin et al,[13] 1998	Number of lacunae: Cerebellum	0=98%; 1-4=2%; 5-9=0%; ≥10=0%.	0=100%; 1-4=0%; 5-9=0%; ≥10=0%.	NS	>0.05	Exact 2 x 4 table test for trend	No	NS	>0.05	Stepwise logistic regression	No
Lin et al,[13] 1998	Number of lacunae: Internal capsule and corona radiata	0=62%; 1-4=30%; 5-9=8%; ≥10=0%.	0=60%; 1-4=38%; 5-9=2%; ≥10=0%.	NS	>0.05	Exact 2 x 4 table test for trend	No	NS	>0.05	Stepwise logistic regression	No
Lin et al,[13] 1998	Frequency of non-lacunar infarcts: left frontal	Present=32% (refer to study for size of infarcts)	Present=12% (refer to study for size of infarcts)	NS	<0.05	Chi-square test	Yes	NS	>0.05	Stepwise logistic regression	No
Lin et al,[13] 1998	Frequency of non-lacunar infarcts: right frontal	Present=12% (refer to study for size of infarcts)	Present=4% (refer to study for size of infarcts)	NS	>0.05	Chi-square test	No	NS	>0.05	Stepwise logistic regression	No
Lin et al,[13] 1998	Frequency of non-lacunar infarcts: left temporal	Present=36% (refer to study for size of infarcts)	Present=4% (refer to study for size of infarcts)	NS	<0.001	Chi-square test	Yes	NS	>0.05	Stepwise logistic regression	No
Lin et al,[13] 1998	Frequency of non-lacunar infarcts: right temporal	Present=8% (refer to study for size of infarcts)	Present=16% (refer to study for size of infarcts)	NS	>0.05	Chi-square test	No	NS	>0.05	Stepwise logistic regression	No
Lin et al,[13] 1998	Frequency of non-lacunar infarcts: left parietal	Present=32% (refer to study for size of infarcts)	Present=2% (refer to study for size of infarcts)	NS	<0.001	Chi-square test	Yes	NS	<0.01	Stepwise logistic regression	Yes
Lin et al,[13] 1998	Frequency of non-lacunar infarcts: right parietal	Present=10% (refer to study for size of infarcts)	Present=18% (refer to study for size of infarcts)	NS	>0.05	Chi-square test	No	NS	>0.05	Stepwise logistic regression	No
Lin et al,[13] 1998	Frequency of non-lacunar infarcts: left occipital	Present=18% (refer to study for size of infarcts)	Present=2% (refer to study for size of infarcts)	NS	>0.05	Chi-square test	No	NS	>0.05	Stepwise logistic regression	No
Lin et al,[13] 1998	Frequency of non-lacunar infarcts: right occipital	Present=6% (refer to study for size of infarcts)	Present=6% (refer to study for size of infarcts)	NS	>0.05	Chi-square test	No	NS	>0.05	Stepwise logistic regression	No
Lin et al,[13] 1998	Frequency of non-lacunar infarcts: left subcortical gray	Present=8% (refer to study for size of infarcts)	Present=6% (refer to study for size of infarcts)	NS	>0.05	Chi-square test	No	NS	>0.05	Stepwise logistic regression	No
Lin et al,[13] 1998	Frequency of non-lacunar infarcts: right subcortical gray	Present=8% (refer to study for size of infarcts)	Present=16% (refer to study for size of infarcts)	NS	>0.05	Chi-square test	No	NS	>0.05	Stepwise logistic regression	No
Lin et al,[13] 1998	Frequency of non-lacunar infarcts: left cortical	Present=66% (refer to study for size of infarcts)	Present=16% (refer to study for size of infarcts)	NS	<0.001	Chi-square test	Yes	NS	>0.05	Stepwise logistic regression	No
Lin et al,[13] 1998	Frequency of non-lacunar infarcts: right cortical	Present=26% (refer to study for size of infarcts)	Present=20% (refer to study for size of infarcts)	NS	>0.05	Chi-square test	No	NS	>0.05	Stepwise logistic regression	No
Loeb et al,[14] 1992	Number of lacunes	Mean=3.65	Mean=3.32	NS	Not significant	t-test	No	N/A	N/A	N/A	N/A

*finding visualised in the harvest plot (Figure 2)
Abbreviations: N/A, not applicable, NS, not stated

eTable 10.6: NUMBER of lesions and post-stroke COGNITIVE IMPAIRMENT

				Unadjusted analysis				Adjusted analysis			
**Study**	**Neuroimaging feature**	**Developed PSCI**	**Not developed PSCI**	**Effect size**	**Sig. level**	**Statistical test**	**Associated with PSCI**	**Effect size**	**Sig. level**	**Statistical test**	**Associated with PSCI**
Schmidt et al,[24] 1993	Number of infarcts - Mean (SD)	Persistent cognitive impairment=1.8 (1.1)	Transient cognitive impairment=1.2 (0.4), No cognitive impairment=1.1 (0.3)	NS	P<0.05 vs controls, P<0.05 vs transient cognitive impairment	Duncan t-test	Yes	N/A	N/A	N/A	N/A
Schmidt et al,[24] 1993	Multiple lesions	Present=50%	Present=16%	NS	P<0.05 vs controls, P<0.01 vs transient cognitive impairment	Duncan t-test	Yes	Beta=1.6; SE (beta)=0.7	<0.05	Stepwise backward regression	Yes
Pinzon et al,[19] 2018	Number of brain lesions	No lesion=20%, Single=31%, Multiple=50%	No lesion=60%, Single=31%, Multiple=9%	Single vs no lesion= OR=2.930 (0.990-8.800), Multiple vs no lesion= OR=17.27 (3.990-86.18)	<0.001	Chi-square test	Yes	Multiple lesions= OR=0.9878 (1.093-89.3140	Multiple lesions= 0.041	Logistic regression	Yes
Sundar et al,[25] 2010	Number of infarcts	Single=38%; Multiple=62%	Single=32%; Multiple=68%	NS	NS	NS	No	N/A	N/A	N/A	N/A

*finding visualised in the harvest plot (Figure 2)
Abbreviations: N/A, not applicable, NS, not stated

eTable 10.7: SWELLING and post-stroke DEMENTIA

				Unadjusted analysis				Adjusted analysis			
**Study**	**Neuroimaging feature**	**Developed PSD/PSCI**	**Not developed PSD/PSCI**	**Effect size**	**Sig. level**	**Statistical test**	**Associated with PSD**	**Effect size**	**Sig. level**	**Statistical test**	**Associated with PSD**
Klimkowicz-Mrowiec et al,[12] 2006	Brain oedema	Present=32%, Absent=68%	Present=21% Absent=79%	NS	0.69	Chi-square	No	N/A	N/A	N/A	N/A

*finding visualised in the harvest plot (Figure 2)
Abbreviations: N/A, not applicable, NS, not stated

Supplement 11: Combinations of neuroimaging features

eTable 11.1: COMBINATIONS of neuroimaging features and post-stroke DEMENTIA

				Unadjusted analysis				Adjusted analysis			
**Study**	**Neuroimaging feature**	**Developed PSD/PSCI**	**Not developed PSD/PSCI**	**Effect size**	**Sig. level**	**Statistical test**	**Associated with PSD**	**Effect size**	**Sig. level**	**Statistical test**	**Associated with PSD**
Mackowiak-Cordoliani et al,[15] 2003	Small vessel disease	Present=24%, Absent=76%	Present=21% Absent=79%	RR=0.84 CI=0.38 – 1.85	>0.05	Kaplan-Meier survival analysis using the log rank test	No	N/A	N/A	N/A	N/A
Rasquin et al,[21] 2004	Presence of SBI or WML	NS	NS	OR=5.6, CI=1.4-22.5	0.01	Chi-square or Fischer's exact test	Yes	OR=7.4, CI=1.1 – 47.2	<0.05	Logistic regression	**Yes**
Appleton et al,[3] 2020	SVD score (of 3), median (IQR)	N/A	N/A	N/A	N/A	N/A	N/A	Refer to study for each cognitive measure	Refer to study for each cognitive measure	Multiple linear regression	Associated with verbal fluency
Appleton et al,[3] 2020	Brain frailty score (/3), median (IQR)	N/A	N/A	N/A	N/A	N/A	N/A	Refer to study for each cognitive measure	Refer to study for each cognitive measure	Multiple linear regression	Associated with t-MMSE, TICS-M, verbal fluency

*finding visualised in the harvest plot (Figure 2)
Abbreviations: N/A, not applicable, NS, not stated

eTable 11.2: COMBINATIONS of neuroimaging features and post-stroke COGNITIVE IMPAIRMENT

				Unadjusted analysis				Adjusted analysis			
**Study**	**Neuroimaging feature**	**Developed PSCI**	**Not developed PSCI**	**Effect size**	**Sig. level**	**Statistical test**	**Associated with PSCI**	**Effect size**	**Sig. level**	**Statistical test**	**Associated with PSCI**
Chausson et al,[7] 2010	Lesions (mainly lacunes and leukoaraiosis)	Present=31%, Absent=69%	Present=21%, Absent=79%	NS	<0.01	The following statistical tests were used, as appropriate= Chi-square test, Fisher exact test, Student t test, or Mann-Whitney U test.	Yes	N/A	N/A	N/A	N/A
House et al,[9] 1990	Presence of Non-stroke pathology (cortical atrophy and white matter attenuation)	NS	NS	OR=3.2, CI=1.1-9.6	<0.05	Two tailed test of significance	Yes	N/A	N/A	N/A	N/A
Rasquin et al,[21] 2004	Presence of SBI or WML	NS	NS	OR=2.3, CI=1.0-5.1	0.05	Chi-square or Fischer's exact test	No	OR=1.5, CI=0.6 – 3.8	>0.05	Logistic regression	**No**

*finding visualised in the harvest plot (Figure 2)
Abbreviations: N/A, not applicable, NS, not stated

Supplement 12: Risk of bias assessment

Risk of bias

Study	D1	D2	D3	D4	D5	D6	Overall
Alexandrova et al, 2016	-	?	+	+	X	-	-
Andersen et al, 1996	-	?	+	+	-	-	-
Appleton et al, 2020	+	-	+	+	+	+	+
Barba et al, 2000	-	-	-	-	-	+	-
Biffi et al, 2016	+	-	+	-	+	+	-
Bornstein et al, 1996	-	+	+	+	X	-	-
Chausson et al, 2010	-	?	+	+	X	-	-
Gomez-Vierra et al, 2002	-	?	+	+	+	+	+
House et al, 1990	-	?	+	+	X	+	-
Jacquin et al, 2014	-	X	+	+	+	+	-
Kase et al, 1998	+	?	-	+	X	+	-
Klimkowicz-Mrowiec et al, 2006	+	X	+	-	+	+	-
Lin et al, 1998	+	?	+	+	+	+	+
Loeb et al, 1992	X	+	+	+	X	-	-
Mackowiak-Cordoliani et al, 2003	-	?	+	-	+	+	-
Mehrabian et al, 2015	-	?	+	+	X	+	-
Miyao et al, 1992	X	+	+	+	X	+	-
Moulin et al, 2016	+	?	+	-	+	+	+
Pinzon et al, 2018	-	+	+	+	+	+	+
Prodjohardjono et al, 2020	-	?	+	+	+	+	+
Rasquin et al, 2004	-	?	X	+	+	+	-
Renjen et al, 2015	-	?	+	+	X	X	-
Saini et al, 2014	-	+	+	+	+	+	+
Schmidt et al, 1993	X	?	-	+	+	+	-
Sundar et al, 2010	-	?	+	-	X	X	-
Tang et al, 2004	+	X	+	+	+	+	-
Thein et al, 2007	-	+	+	+	X	+	-
Zhang et al, 2012	-	-	-	+	+	+	-

D1: Study participation
D2: Study attrition
D3: Prognostic factor measurement
D4: Outcome measurement
D5: Adjustment for other prognostic factors
D6: Statistical analysis and reporting

Judgement
X High
- Moderate
+ Low
? No information

We used the QUIPS tool to assess: study participation, study attrition, prognostic factor measurement, outcome measurement, adjustment for other prognostic factors, statistical analysis and reporting. Overall risk of bias was calculated by summing the rating for each risk of bias domain (low=1, moderate=2, high=3) and categorising the overall risk of bias into (low=1-7, moderate=8-13, high=14-18). When risk of bias domains were scored as unclear, the authors used their discretion to decide whether the overall risk of bias rating should be increased. The risk of bias plot was created using the Risk-of-bias VISualization (robvis) web application.[42]

Supplement 13: GRADE assessment

№ of studies	Certainty assessment						Certainty
	Study design	Risk of bias	Inconsistency	Indirectness	Imprecision	Other considerations	
**PRESENCE of cerebral atrophy and post-stroke DEMENTIA**							
4[12, 13, 18, 21]	observational studies	not serious	not serious	not serious	serious [a]	none	⨁⨁⨁◯ MODERATE
**PRESENCE of WML and post-stroke DEMENTIA**							
6[12, 13, 17, 18, 21, 26]	observational studies	not serious	serious [b,c]	not serious	very serious [a,c]	none	⨁◯◯◯ VERY LOW
**PRESENCE of pre-existing stroke lesions and post-stroke DEMENTIA**							
3[6, 15, 21]	observational studies	not serious	not serious	not serious	very serious [a,c]	none	⨁⨁◯◯ LOW
**ISCHAEMIC STROKE (vs. haemorrhagic stroke) and post-stroke DEMENTIA**							
4[,7,9,10]	observational studies	not serious	not serious	not serious	serious [a]	none	⨁⨁⨁◯ MODERATE
**PRESENCE of cerebral atrophy and post-stroke COGNITIVE IMPAIRMENT**							
3[8, 21, 24]	observational studies	not serious	serious [b]	not serious	very serious [a,c]	none	⨁◯◯◯ VERY LOW
**PRESENCE of WML and post-stroke COGNITIVE IMPAIRMENT**							
4[10, 21, 24, 27]	observational studies	not serious	not serious	not serious	serious [a]	none	⨁⨁⨁◯ MODERATE
**PRESENCE of pre-existing stroke lesions and post-stroke COGNITIVE IMPAIRMENT**							
2[10, 21]	observational studies	not serious	not serious	not serious	very serious [a,c]	none	⨁⨁◯◯ LOW
**ISCHAEMIC STROKE (vs. haemorrhagic stroke) and post-stroke COGNITIVE IMPAIRMENT**							
3[10, 22, 28]	observational studies	not serious	serious [c]	not serious	serious [a]	none	⨁⨁◯◯ LOW

Explanations:

a. Downgraded due to wide confidence intervals
b. Downgraded because test for heterogeneity $p<0.05$
c. Downgraded due to large I squared (≥41%)

Created using the GRADEpro Guideline Development Tool[43]

Supplement 14: Sensitivity Analysis PSD

**PSD Studies**	**Patients without pre-stroke dementia and/or cognitive impairment only**				**Patients with ischaemic stroke only**				**PSD diagnosed at least six months after the index stroke only**			
**CT feature**	**No. of studies**	**No. of patients**	**OR (95% CI)**	**Sig. of pooled OR**	**No. of studies**	**No. of patients**	**OR (95% CI)**	**Sig. of pooled OR**	**No. of studies**	**No. of patients**	**OR (95% CI)**	**Sig. of pooled OR**
Atrophy	4[12, 13, 18, 21]	558	OR=2.80, 95% CI=1.21-6.51	p<.02	3[12, 13, 21]	323	OR=2.21, 95% CI=0.83-5.86	p=0.11	2[18, 21]	264	OR=6.27, 95% CI=2.17-18.11	p<.001
WML	5[12, 13, 17, 18, 21]	775	OR=3.11, 95% CI=1.42-6.80	p=.005	4[12, 13, 17, 21]	538	OR=2.98, 95% CI=1.05-8.47	p=.04	3[17, 18, 21]	480	OR=3.79, 95% CI=1.01-14.22	p=.05
Pre-existing stroke lesions	3[6, 15, 21]	352	OR=2.38, 95% CI=1.06-5.32	p=.04	1[21]	Insufficient number of studies			3[6, 15, 21]	352	OR=2.38, 95% CI=1.06-5.32	p=.04
Ischemic stroke (vs haemorrhagic stroke)	3[4, 12, 15]	590	OR=1.05, 95% CI=0.56-1.96	p=.88	N/A				1[15]	Insufficient number of studies		

Supplement 15: Sensitivity Analysis PSCI

PSCI Studies	Patients without pre-stroke dementia and/or cognitive impairment only				Patients with ischaemic stroke only				PSCI diagnosed at least six months after the index stroke only			
**CT feature**	**No. of studies**	**No. of patients**	**OR (95% CI)**	**Sig. of pooled OR**	**No. of studies**	**No. of patients**	**OR (95% CI)**	**Sig. of pooled OR**	**No. of studies**	**No. of patients**	**OR (95% CI)**	**Sig. of pooled OR**
Atrophy	3[8, 21, 24]	501	OR=2.03, 95% CI=0.74-5.56	p=.17	3[8, 21, 24]	501	OR=2.03, 95% CI=0.74-5.56	p=.17	3[8, 21, 24]	501	OR=2.03, 95% CI=0.74-5.56	p=.17
WML	3[10, 21, 24]	413	OR=3.45, 95% CI=2.04-5.82	p<.001	3[21, 24, 27]	260	OR=3.99, 95% CI=2.01-7.94	p<.001	2[21, 24]	200	OR=4.32, 95% CI=1.78-10.46	p=.001
Pre-existing stroke lesions	2[10, 21]	379	OR=2.42, 95% CI=0.89-6.56	p=.08	1[21]	Insufficient number of studies			1[21]	Insufficient number of studies		
Ischemic stroke (vs haemorrhagic stroke)	2[10, 28]	797	OR=1.04, 95% CI=0.34-3.13	p=.95	N/A				1[22]	Insufficient number of studies		

Supplement 16: Meta-analysis of risk factors associated with PSD (unadjusted prognostic factors)

Risk factors	No. of studies	No. of patients	OR (95% CI)	Sig. of pooled OR	I squared	p-value for heterogeneity
**CT Neuroimaging features**						
Atrophy	4 [12, 13, 18, 21]	588	**2.80 (1.21-6.51)**	**0.02**	38%	0.18
WML	6 [12, 13, 17, 18, 21, 26]	1054	**2.46 (1.25-4.84)**	**0.009**	73%	0.009
Pre-existing stroke lesions	3 [6, 15, 21]	352	**2.38 (1.06-5.32)**	**0.04**	52%	0.13
Ischemic stroke (vs haemorrhagic stroke)	4 [4, 12, 15, 26]	869	1.06 (0.62-1.82)	0.83	0%	0.90
**Demographic factors**						
Female	7 [4, 6, 12, 13, 15, 18, 21]	1116	**1.58 (1.03-2.43)**	**0.04**	82%	<0.001
Low education	5 [4, 12, 15, 18, 21]	854	1.97 (0.93-4.19)	0.08	22%	0.27
**Vascular factors**						
Hypertension	5 [4, 12, 15, 18, 26]	1087	**1.43 (1.02-2.00)**	**0.04**	0%	0.64
Diabetes	5 [4, 12, 15, 18, 26]	1087	**2.13 (1.22-3.70)**	**0.008**	62%	0.03
Atrial fibrillation	3 [4, 12, 26]	725	**2.23 (1.04-4.79)**	**0.04**	56%	0.10
Hypercholesterolemia	2 [15, 18]	362	1.00 (0.40-2.48)	>0.99	63%	0.10
Smoking	5 [4, 12, 15, 18, 26]	1087	**0.57 (0.33-0.98)**	**0.04**	65%	0.02
High alcohol intake	3 [4, 15, 18]	613	**0.54 (0.35-0.83)**	**0.005**	0%	0.57
Ischemic heart disease	4 [4, 12, 18, 26]	943	1.36 (0.81-2.28)	0.24	1%	0.39
Prior stroke	4 [4, 12, 15, 26]	869	**1.66 (1.13-2.46)**	**0.01**	0%	>0.99
Prior TIA	4 [4, 12, 15, 26]	869	1.03 (0.49-2.17)	0.93	0%	0.93
Stroke Severity	1 [26]	Insufficient number of studies				
APOE	0	Insufficient number of studies				

*Bold values indicate statistically significant result

Supplement 17: Meta-analysis of risk factors associated with PSCI (unadjusted prognostic factors)

Risk factors	No. of studies	No. of patients	OR (95% CI)*	Sig. of pooled OR*	I squared	p-value for heterogeneity
**CT Neuroimaging features**						
Atrophy	3[8, 21, 24]	501	2.03 (0.74-5.56)	0.17	68%	0.04
WML	4[10, 21, 24, 27]	473	**3.46 (2.17-5.52)**	**<0.001**	0%	0.93
Pre-existing stroke lesions	2[10, 21]	379	2.42 (0.89-6.56)	0.08	71%	0.06
Ischemic stroke (vs haemorrhagic stroke)	3[10, 22, 28]	847	0.84 (0.32-2.18)	0.72	53%	0.12
**Demographic factors**						
Female	9[1, 8, 10, 19, 21-24, 28]	1823	**1.54 (1.01-2.34)**	**0.04**	50%	0.04
Low education	8[1, 8, 10, 19, 21, 22, 25, 28]	1628	**1.95 (1.22-3.13)**	**0.005**	71%	<0.001
**Vascular factors**						
Hypertension	7[1, 8, 10, 19, 24, 25, 28]	1460	1.36 (0.87-2.15)	0.18	62%	0.01
Diabetes	7[1, 8, 10, 19, 24, 25, 28]	1460	**3.24 (1.04-10.03)**	**0.04**	89%	<0.001
Atrial fibrillation	3[8, 10, 28]	1098	**1.84 (1.10-3.08)**	**0.02**	0%	0.53
Hypercholesterolemia	6[8, 10, 19, 24, 25, 28]	1413	0.98 (0.72-1.35)	0.91	15%	0.32
Smoking	6[1, 8, 10, 19, 24, 25]	883	0.75 (0.53-1.08)	0.13	4%	0.39
High alcohol intake	3[1, 8, 10]	568	1.01 (0.58-1.76)	0.98	0%	0.65
Ischemic heart disease	2[8, 10]	521	1.29 (0.53-3.15)	0.58	21%	0.26
Prior stroke	3[1, 8, 19]	458	0.91 (0.25-3.25)	0.88	88%	<0.001
Prior TIA	2[8, 10]	521	1.56 (0.96-2.52)	0.07	0%	0.70
Stroke Severity	2[23, 28]	895	**1.16 (1.04-1.30)**	**0.01**	0%	0.56
APOE	0	Insufficient number of studies				

*Bold values indicate statistically significant result

References

1. Alexandrova ML and Danovska MP. Cognitive impairment one year after ischemic stroke: predictorsand dynamics of significant determinants. *Turkish journal of medical sciences* 2016; 46: 1366-1373.
2. Andersen G, Vestergaard K, Riis JØ, et al. Intellectual impairment in the first year following stroke, compared to an age-matched population sample. *Cerebrovasc Dis* 1996; 6: 363-369.
3. Appleton JP, Woodhouse LJ, Adami A, et al. Imaging markers of small vessel disease and brain frailty, and outcomes in acute stroke. *Neurology* 2020; 94: e439-e452. DOI: https://dx.doi.org/10.1212/WNL.0000000000008881.
4. Barba R, Martinez-Espinosa S, Rodriguez-Garcia E, et al. Poststroke dementia: Clinical features and risk factors. *Stroke* 2000; 31: 1494-1501. DOI: http://dx.doi.org/10.1161/01.STR.31.7.1494.
5. Biffi A, Bailey D, Anderson CD, et al. Risk factors associated with early vs delayed dementia after intracerebral hemorrhage. *JAMA Neurology* 2016; 73: 969-976. DOI: http://dx.doi.org/10.1001/jamaneurol.2016.0955.
6. Bornstein NM, Gur AY, Treves TA, et al. Do silent brain infarctions predict the development of dementia after first ischemic stroke? *Stroke* 1996; 27: 904-905.
7. Chausson N, Olindo Sp, Cabre P, et al. Five-year outcome of a stroke cohort in Martinique, French West Indies: Etude Realisee en Martinique et Centree sur l'Incidence des Accidents vasculaires cerebraux, Part 2. *Stroke* 2010; 41: 594-599.
8. Gomez-Viera N, Martin-Labrador M, Guevara-Ferrer M, et al. Prognostic factors of cognitive deterioration in patients with cerebral infarcts. [Spanish]. *Rev Neurol* 2002; 34: 223-231.
9. House A, Dennis M, Warlow C, et al. The relationship between intellectual impairment and mood disorder in the first year after stroke. *Psychological Medicine* 1990; 20: 805-814.
10. Jacquin A, Binquet C, Rouaud O, et al. Post-stroke cognitive impairment: High prevalence and determining factors in a cohort of mild stroke. *Journal of Alzheimer's Disease* 2014; 40: 1029-1038. DOI: http://dx.doi.org/10.3233/JAD-131580.
11. Kase C, Wolf P, Kelly-Hayes M, et al. Intellectual decline after stroke: the Framingham Study. *Stroke* 1998; 29: 805-812.
12. Klimkowicz-Mrowiec A, Dziedzic T, Słowik A, et al. Predictors of poststroke dementia: results of a hospital-based study in Poland. *Dementia and geriatric cognitive disorders* 2006; 21: 328-334.
13. Lin RT, Lai CL, Tai CT, et al. Cranial computed tomography in ischemic stroke patients with and without dementia--a prospective study. *Kaohsiung J Med Sci* 1998; 14: 203-211.
14. Loeb C, Gandolfo C, Croce R, et al. Dementia associated with lacunar infarction. *Stroke* 1992; 23: 1225-1229.
15. Mackowiak-Cordoliani M-A, Hénon H, Pruvo J-P, et al. Poststroke dementia: influence of hippocampal atrophy. *Archives of neurology* 2003; 60: 585-590.
16. Mehrabian S, Raycheva M, Petrova N, et al. Neuropsychological and neuroimaging markers in prediction of cognitive impairment after ischemic stroke: A prospective follow-up study. *Neuropsychiatric Disease and Treatment Vol 11 2015, ArtID 2711-2719* 2015; 11.

17. Miyao S, Takano A, Teramoto J, et al. Leukoaraiosis in relation to prognosis for patients with lacunar infarction. *Stroke* 1992; 23: 1434-1438.
18. Moulin S, Labreuche J, Bombois S, et al. Dementia risk after spontaneous intracerebral haemorrhage: A prospective cohort study. *The Lancet Neurology* 2016; 15: 820-829. DOI: http://dx.doi.org/10.1016/S1474-4422%2816%2900130-7.
19. Pinzon RT, Sanyasi RDL and Totting S. The prevalence and determinant factors of post-stroke cognitive impairment. *Asian Pacific J Heal Sci* 2018; 5: 78-83.
20. Prodjohardjono A, Vidyanti AN, Susianti NA, et al. Higher level of acute serum VEGF and larger infarct volume are more frequently associated with post-stroke cognitive impairment. *PLoS ONE* 2020; 15: e0239370. DOI: https://dx.doi.org/10.1371/journal.pone.0239370.
21. Rasquin SM, Verhey FR, van Oostenbrugge RJ, et al. Demographic and CT scan features related to cognitive impairment in the first year after stroke. *Journal of Neurology, Neurosurgery & Psychiatry* 2004; 75: 1562-1567.
22. Renjen PN, Gauba C and Chaudhari D. Cognitive impairment after stroke. *Cureus* 2015; 7.
23. Saini M, Tan CS, Hilal S, et al. Computer tomography for prediction of cognitive outcomes after ischemic cerebrovascular events. *J Stroke Cerebrovasc Dis* 2014; 23: 1921-1927. DOI: https://dx.doi.org/10.1016/j.jstrokecerebrovasdis.2014.02.007.
24. Schmidt R, Mechtler L, Kinkel PR, et al. Cognitive impairment after acute supratentorial stroke: a 6-month follow-up clinical and computed tomographic study. *European archives of psychiatry and clinical neuroscience* 1993; 243: 11-15.
25. Sundar U and Adwani S. Post-stroke cognitive impairment at 3 months. *Ann* 2010; 13: 42.
26. Tang WK, Chan SS, Chiu HF, et al. Frequency and determinants of poststroke dementia in Chinese. *Stroke* 2004; 35: 930-935.
27. Thein SS, Hamidon BB, Teh HS, et al. Leukoaraiosis as a predictor for mortality and morbidity after an acute ischaemic stroke. *Singapore Med J* 2007; 48: 396-399.
28. Zhang Y, Zhang Z, Yang B, et al. Incidence and risk factors of cognitive impairment 3 months after first-ever stroke: a cross-sectional study of 5 geographic areas of China. *Journal of Huazhong University of Science and Technology [Medical Sciences]* 2012; 32: 906-911.
29. Ankolekar S, Renton C, Sare G, et al. Relationship between poststroke cognition, baseline factors, and functional outcome: data from "efficacy of nitric oxide in stroke" trial. *J Stroke Cerebrovasc Dis* 2014; 23: 1821-1829. DOI: https://dx.doi.org/10.1016/j.jstrokecerebrovasdis.2014.04.022.
30. Benedictus MR, Hochart A, Rossi C, et al. Prognostic Factors for Cognitive Decline After Intracerebral Hemorrhage. *Stroke* 2015; 46: 2773-2778. DOI: https://dx.doi.org/10.1161/STROKEAHA.115.010200.
31. Cordonnier C, Hénon H, Derambure P, et al. Early epileptic seizures after stroke are associated with increased risk of new-onset dementia. *Journal of Neurology, Neurosurgery & Psychiatry* 2007; 78: 514-516.
32. del Ser T, Barba R, Morin MM, et al. Evolution of cognitive impairment after stroke and risk factors for delayed progression. *Stroke* 2005; 36: 2670-2675.
33. Hénon H, Durieu I, Guerouaou D, et al. Poststroke dementia. *Incidence and relationship to prestroke cognitive decline* 2001; 57: 1216-1222. DOI: 10.1212/wnl.57.7.1216.

34. Mok V, Chang C, Wong A, et al. Neuroimaging determinants of cognitive performances in stroke associated with small vessel disease. *J Neuroimaging* 2005; 15: 129-137.
35. Pasquier F, Henon H and Leys D. Relevance of white matter changes to pre- and poststroke dementia. *Ann N Y Acad Sci* 2000; 903: 466-469. DOI: http://dx.doi.org/10.1111/j.1749-6632.2000.tb06400.x.
36. Rasquin SMC, Lodder J and Verhey FRJ. Predictors of reversible mild cognitive impairment after stroke: A 2-year follow-up study. *Journal of the Neurological Sciences* 2005; 229-230: 21-25. DOI: http://dx.doi.org/10.1016/j.jns.2004.11.015.
37. Rasquin SMC, Van Oostenbrugge RJ, Verhey FRJ, et al. Vascular mild cognitive impairment is highly prevalent after lacunar stroke but does not increase over time: A 2-year follow-up study. *Dementia and Geriatric Cognitive Disorders* 2007; 24: 396-401. DOI: http://dx.doi.org/10.1159/000109747.
38. Sharpe M, Hawton K, House A, et al. Mood disorders in long-term survivors of stroke: associations with brain lesion location and volume. *Psychological medicine* 1990; 20: 815-828.
39. Tang WK, Chan SS, Chiu HF, et al. Frequency and clinical determinants of poststroke cognitive impairment in nondemented stroke patients. *Journal of geriatric psychiatry and neurology* 2006; 19: 65-71.
40. Tang WK, Chan SS, Chiu HF, et al. Impact of applying NINDS-AIREN criteria of probable vascular dementia to clinical and radiological characteristics of a stroke cohort with dementia. *Cerebrovasc Dis* 2004; 18: 98-103.
41. Xiong L, Charidimou A, Pasi M, et al. Predictors for Late Post-Intracerebral Hemorrhage Dementia in Patients with Probable Cerebral Amyloid Angiopathy. *Journal of Alzheimer's Disease* 2019; 71: 435-442. DOI: https://dx.doi.org/10.3233/JAD-190346.
42. McGuinness LA and Higgins JPT. Risk-of-bias VISualization (robvis): An R package and Shiny web app for visualizing risk-of-bias assessments. *Research Synthesis Methods* 2020; n/a. DOI: 10.1002/jrsm.1411.
43. GRADEpro GDT: GRADEpro Guideline Development Tool [Software]. McMaster University, 2020 (developed by Evidence Prime, Inc.). Available from gradepro.org.
